# Tuberculosis vaccines and therapeutic drug: challenges and future directions

**DOI:** 10.1186/s43556-024-00243-6

**Published:** 2025-01-22

**Authors:** Yajing An, Ruizi Ni, Li Zhuang, Ling Yang, Zhaoyang Ye, Linsheng Li, Seppo Parkkila, Ashok Aspatwar, Wenping Gong

**Affiliations:** 1 Beijing Key Laboratory of New Techniques of Tuberculosis Diagnosis and Treatment, Senior Department of Tuberculosis, The Eighth Medical Center of PLA General Hospital, 17#Heishanhu Road, Haidian District, Beijing, 100091 China; 2 Graduate School, Hebei North University, Zhangjiakou, 075000 Hebei China; 3 Faculty of Medicine and Health Technology, Tampere University, 33014 Tampere, Finland; 4 Department of Clinical Chemistry, Fimlab Laboratories PLC, Tampere, Finland

**Keywords:** *Mycobacterium tuberculosis* (MTB), Tuberculosis (TB), Antigens, Therapeutic drug, Host-directed therapies, Multidrug-resistance (MDR)

## Abstract

Tuberculosis (TB) remains a prominent global health challenge, with the World Health Organization documenting over 1 million annual fatalities. Despite the deployment of the Bacille Calmette-Guérin (BCG) vaccine and available therapeutic agents, the escalation of drug-resistant *Mycobacterium tuberculosis* strains underscores the pressing need for more efficacious vaccines and treatments. This review meticulously maps out the contemporary landscape of TB vaccine development, with a focus on antigen identification, clinical trial progress, and the obstacles and future trajectories in vaccine research. We spotlight innovative approaches, such as multi-antigen vaccines and mRNA technology platforms. Furthermore, the review delves into current TB therapeutics, particularly for multidrug-resistant tuberculosis (MDR-TB), exploring promising agents like bedaquiline (BDQ) and delamanid (DLM), as well as the potential of host-directed therapies. The hurdles in TB vaccine and therapeutic development encompass overcoming antigen diversity, enhancing vaccine effectiveness across diverse populations, and advancing novel vaccine platforms. Future initiatives emphasize combinatorial strategies, the development of anti-TB compounds targeting novel pathways, and personalized medicine for TB treatment and prevention. Despite notable advances, persistent challenges such as diagnostic failures and protracted treatment regimens continue to impede progress. This work aims to steer future research endeavors toward groundbreaking TB vaccines and therapeutic agents, providing crucial insights for enhancing TB prevention and treatment strategies.

**Supplementary Information:**

The online version contains supplementary material available at 10.1186/s43556-024-00243-6.

## Introduction

Tuberculosis (TB) continues to be a leading cause of infectious disease-related mortality worldwide, with the World Health Organization's 2023 Global Tuberculosis Report estimating 1.3 million TB-related deaths in 2022. Globally, over 10 million new cases of active TB are reported annually, highlighting the disease's ongoing public health challenge [1–4]. In high-burden countries like China, which reported 748,000 new cases and 30,000 deaths in 2022, TB remains a significant concern due to its widespread prevalence and the emergence of drug-resistant strains of *Mycobacterium tuberculosis* (MTB) [5].

Vaccination has been proven to be one of the most cost-effective public health strategies [6], playing a crucial role in achieving the World Health Organization's (WHO) global strategy to end TB by 2035 [7]. Currently, the Bacille Calmette-Guérin (BCG) vaccine remains the only approved vaccine for TB prevention [8–10]. Despite its critical role in reducing severe TB in infants and children, BCG's limited efficacy in preventing adult pulmonary TB and its ineffectiveness against primary or latent TB infection highlight the urgent need for more effective vaccines [4, 11–13]. With the continuous exploration and in-depth research in TB vaccinology, numerous new vaccine candidates are currently in various stages of clinical trials, while many more are at the preclinical stage. The primary aim is to either replace the BCG vaccine or to enhance its efficacy through a prime-boost immunization strategy [14]. Furthermore, recent advances in bioinformatics and artificial intelligence (AI) have significantly transformed vaccine research, particularly in the identification of novel MTB antigens and vaccine targets [15–17]. AI can be used in antigen discovery and vaccine design by analyzing large datasets from various sources to identify patterns and predict potential antigens that could be effective in vaccine development [18]. It can predict the immunogenicity and antigenicity of candidate antigens, model the three-dimensional structures of proteins, and perform molecular docking simulations [19, 20]. AI also expedites the screening of a vast number of potential antigens by automating the analysis of experimental data [21, 22]. Furthermore, it revolutionizes the development of personalized vaccines by analyzing individual genetic and immunological profiles, and integrates diverse data types to uncover new insights into the pathogenesis of TB and the immune responses to infection.

The management of TB is pivotal in curbing the spread of this pervasive disease; however, therapeutic advancements in combating TB are impeded by a myriad of developmental challenges. Anti-tuberculosis pharmaceuticals are bifurcated into two distinct tiers. The first-line regimen predominantly comprises isoniazid, rifamycins, pyrazinamide, and ethambutol, which are the cornerstone of therapy for non-drug-resistant and drug-sensitive TB cases. In contrast, second-line agents, encompassing levofloxacin, moxifloxacin, bedaquiline (BDQ), and delamanid (DLM), are invariably resorted to in scenarios where drug resistance is established [23–26]. While new drugs like BDQ and DLM offer hope for treating multidrug-resistant tuberculosis (MDR-TB), issues such as diagnostic failures, lengthy treatment regimens, and adverse side effects pose significant hurdles [27, 28]. Current therapies focus on both drug-sensitive and latent TB infections, but new strategies are essential to enhance treatment efficacy and patient outcomes.

This review aims to provide a comprehensive overview of the current state of TB vaccine and therapeutic drug development. By examining recent advances and ongoing research, it highlights the potential for novel vaccines and therapeutic strategies to transform TB prevention and treatment, ultimately contributing to global efforts to end the TB epidemic.

## Antigen selection for TB vaccines

The selection of antigens is a crucial step in the development of effective TB vaccines. This process involves identifying components that can elicit a strong and protective immune response. The advancement of bioinformatics and AI has significantly enhanced our ability to identify and evaluate potential TB antigens.

### Overview of TB antigen categories

With the rapid advancement of genomics and proteomics, research on MTB has entered the post-genomic era. Since the completion of the full genome sequencing of the MTB H37Rv strain by the Sanger Center and the Pasteur Institute in 1998, studies on MTB antigens have deepened, laying an important foundation for the prevention, diagnosis, and treatment of TB [29–33].

MTB antigens can be categorized based on their biological characteristics, physicochemical properties, and functions. The main categories of MTB antigens include [34]: (1) Cell wall and capsular antigens: MTB's cell wall and capsule contain glycolipids, lipoproteins, and glycoproteins such as cord factor and lipoarabinomannan [35]. These antigens are key for immune activation and vaccine development. (2) Secreted antigens: MTB secretes proteins like Ag85A/B and ESAT-6, which can modulate the host's immune system [36, 37]. These antigens are strong candidates for subunit vaccines due to their immunogenicity and ease of production. (3) Dormancy phase antigens: During dormancy, MTB relies on antigens regulated by the DosR regulon, such as HspX, for survival. These antigens are promising for vaccines targeting latent TB infection [38]. (4) Resuscitation phase antigens: Resuscitation promotion factors (Rpfs) like RpfB are involved in reactivating dormant MTB and are potential antigens for vaccines addressing LTBI (latent tuberculosis infection) [39, 40]. (5) BCG regions of difference (RD) antigens: BCG strains lack 16 RD antigens found in MTB, including ESAT-6, which are crucial for pathogenicity [4, 18]. These antigens are important for developing more effective vaccines. In this review, we have compiled a list of 84 MTB antigens that show potential or have demonstrated promising experimental results in TB vaccine development (Table 1).

**Table 1:** Immunological characteristics of 84 MTB antigens: potential targets for novel vaccine development

Antigen (Gene)	Length (aa)	Location ^a^	Phase	Antigenicity ^b^	Immunogenicity ^c^	Hydrophilicity ^d^	Allergenicity ^e^	Toxicity ^f^	Function	Vaccines containing the antigens in preclinical and clinical trials	Interacting Proteins
Rv1738	94	C, M	Latent	Moderate	High	+	‒	‒	Unknown	No	acg, Rv3134c, hspX, hrp1, Rv3127
Rv2450c (rpfE)	172	E	Latent	High	High	+	‒	‒	Stimulation of dormant cell revival, peptidoglycan hydrolase activity	It performs well in preclinical trials and may be a good candidate vaccine	ripA, Rv1954A, Rv2449c, cwlM, Rv2451
Rv2623 (TB31.7)	297	C, CW, M	Latent	Low	High	‒	‒	‒	May play a role in establishing persistent infection	No	hspX, hrp1, devR, Rv1738, acg
Rv1009 (rpfB)	362	E, M	Latent	Moderate	High	‒	‒	‒	Involved in the resuscitation of dormant cells	MVATG18598	ksgA, ripA, tatD, fbpB, vapB47
Rv0867c (rpfA)	407	E	Latent	Moderate	High	+	‒	‒	Involved in the resuscitation of dormant cells	rBCG AERAS-407	Rv1954A, crp, ripA, whiB1, serC
Rv2031c (HspX)	144	C, E, CW, M	Latent	Very High	High	+	+	‒	Chaperone, may play a role in the virulence of MTB	TB/FLU-05E	Rv2030c, TB31.7, acg, Rv1738, hrp1
Rv1886c (Ag85B, fbpB, mpt59)	325	E, CW, M	Early	Moderate	Low	+	‒	‒	Cell wall biogenesis, immune response	AEC/BC02, H56:IC31, MVATG18598, ID91 repRNA、rBCG AERAS-407	fbpC, Rv1885c, fbpA, esxA, mpt64
Rv0288 (TB10.4, esxH)	96	C, E, P	Early	Moderate	Moderate	+	‒	‒	Disruption of ESCRT function and promotion of bacterial growth	TB/FLU-05E, MVATG18598	esxG, espG3, esxB, esxA, PPE4
Rv2032 (acg)	331	C, CW, M	Latent	Low	High	+	‒	‒	Unknown	It performs well in preclinical trials and may be a good candidate vaccine	Rv1738, hspX, Rv2030c, Rv1733c, Rv3134c
Rv2626c (hrp1)	143	C, E, CW, M	Latent	Low	High	‒	‒	‒	Unknown	MVATG18598	Rv1738, Rv1733C, hspX, TB31.7, Rv2030c
Rv3873 (PPE68)	368	CS, ER, CM	Latent	High	Low	+	‒	‒	Immune modulation, interacts with human TLR2, stimulates IL-10 and MCP-1 secretion	H107e/CAF®10b	PE35, esxB, espG1, espJ, esxA
Rv2005c	295	CW, CM	Latent	Moderate	High	‒	+	‒	May be associated with aminoglycoside drug resistance and virulence	No	Rv2004c, Rv2003c, otsB1, devR, devS
Rv3127	344	CW, CM	Latent	Moderate	High	+	‒	‒	NA	No	Rv1738, Rv3134c, Rv1996, tgs1, Rv3131
Rv1733c	210	CM	Latent	Moderate	High	‒	‒	‒	Strong T cell and IFN-γ inducer, potential vaccine candidate	rBCGΔureC∷hly	Rv3134c, hrp1, acg, hspX, Rv1738
Rv1996	317	CW, CM	Latent	Moderate	High	‒	‒	‒	NA	No	Rv0079, Rv1738, Rv3131, hspX, Rv3127
Rv2389c (rpfD)	154	ER, CM	Latent	High	Moderate	‒	‒	‒	Stimulation of dormant cell revival, PG hydrolase activity	MVATG18598, ID91 repRNA, rBCG AERAS-407	Rv2390c, Rv1954A, hemN, ripA, PE_PGRS20
Rv0685 (tuf)	396	Cic	NA	Low	High	+	‒	‒	Promotes GTP-dependent binding of aminoacyl-tRNA to the ribosome A site	No	rpsL, rpsG, rpsJ, rpIB, rpsE
Rv2628	120	NA	Latent	Moderate	High	+	‒	‒	Strong T cell and IFN-γ inducer, potential vaccine candidate	It performs well in preclinical trials and may be a good candidate vaccine	hrp1, Rv1733c, ctpF, Rv2624c, Rv2627c
Rv1980c (mpt64)	228	ER, CW	Latent	High	Low	+	+	+	NA	H107e/CAF®10b	fbpB, esxA, mpt63, esxB, nrdF1
Rv3804c (Ag85A, fbpA, mpt44)	338	C, ER, CW, CM	Early	Moderate	Low	+	‒	‒	Cell wall biogenesis, immune response, involved in the formation of TDM	GamTBvac, Ad5Ag85A, TB/FLU-04L, ChAdOx1 85A, MVA85A、rBCG AERAS-407	fbpC, fbpB, lipY, dagK, esxA
Rv0079	273	C, CW, M	Latent	Moderate	High	+	‒	‒	Translation regulation, immune modulation	No	Rv0080, Rv3134c, Rv1738, rpsl, Rv1996
Rv3130c (tgs1)	463	CW, M	Latent	Moderate	High	+	‒	‒	Triacylglycerol synthesis, energy storage	No	Rv3131, hspX, acg, lipY, Rv2627c
Rv3131	344	C, M	Latent	Moderate	High	+	‒	‒	Immune modulation, potential involvement in innate immune response	It performs well in preclinical trials and may be a good candidate vaccine	tgs1, Rv3134c, Rv1738, fdxA, Rv2030c
Rv0824c (desA1)	338	CS, C, CW, M	NA	Moderate	Moderate	+	‒	‒	Fatty acid biosynthesis, desaturation	No	desA2, dus, desA3, fas, Rv3230c
Rv1908c (katG)	740	C, E, CW, M	NA	Moderate	High	+	+	NA	Detoxification of reactive oxygen species, contributes to drug resistance	No	furA, sodA, ahpC, pncA, embB
Rv1174c (TB8.4)	110	E, CW	NA	Moderate	Low	‒	‒	‒	Immune response, potential T-cell antigen	No	esxH, esxB, esxA, espC, Rv3165c
Rv1349 (irtB)	579	C, M	NA	Moderate	Very High	‒	‒	NA	Iron acquisition, essential for intracellular replication	No	irtA, mbtB, mbtA, mbtE, mbtl
Rv1813c	143	NA	Latent	High	High	+	‒	‒	Unknown, potential vaccine target	ID93 + GLA-SE, MVATG18598	Rv2030c, acg, Rv3127, Rv1733c, fdxA
Rv2006	1327	E, CW, M	Latent	Moderate	Very High	+	‒	NA	Immune response, potential vaccine candidate	It performs well in preclinical trials and may be a good candidate vaccine	treS, treZ, otsB2, Rv2402, otsA
Rv2029c (pfkB)	339	C	Latent	Moderate	High	‒	‒	‒	Glycolysis, essential for energy production	It performs well in preclinical trials and may be a good candidate vaccine	Rv2030c, Rv2028c, pfkA, fba, pgi
Rv2627c	413	M	Latent	Low	High	+	‒	‒	T cell and IFN-γ induction, potential vaccine candidate	It performs well in preclinical trials and may be a good candidate vaccine	tgs1, Rv1733c, TB31.7, acg, narK2
Rv2780 (ald)	371	C, E, CW, M	NA	Moderate	High	‒	‒	‒	Catalyzes the reversible reduction of pyruvate to L-alanine	No	alr, aspC, pntB, Rv2779c, gItB
Rv1884c (rpfC)	176	E	Latent	Moderate	Moderate	+	‒	‒	Involved in the resuscitation of dormant cells	rBCG AERAS-407	Rv1883c, Rv1954A, Rv1885c, Rv1815, fbpB
Rv2620c	141	M	NA	High	High	‒	‒	‒	Unknown	No	Rv2619c, Rv2621c, Rv2622, fdxA, TB31.7
Rv2744c (pspA)	270	C, CW, M	NA	High	Low	+	‒	‒	Involved in stress response and promoting intracellular growth of MTB	No	clgR, Rv2743c, sigB, htpX, Rv2468c
Rv3875 (esxA, ESAT-6)	95	C, E	Early	Moderate	Moderate	+	‒	‒	Multifunctional roles in immune modulation and bacterial escape	AEC/BC02, GamTBvac, TB/FLU-04L, H56:IC31, H107e/CAF®10b, MVATG18598	esxB, eccD1, eccCb1, espl, fbpB
Rv2030c	681	C, CW, M	Latent	Low	Very High	+	‒	NA	Unknown	No	pfkB, Rv2028c, hspX, acg, rip3
Rv3132c (devS, dosS)	578	C, CW, M	Latent	Moderate	High	‒	‒	NA	Part of two-component regulatory system involved in dormancy response	No	devR, Rv3134c, Rv0569, Rv2028c, narL
Rv3347c (PPE55)	3157	NA	NA	High	Very High	‒	‒	NA	Unknown	No	PPE8, PPE56, cpnT, csm5, Rv2082
Rv0467 (icl)	428	C, M	NA	Moderate	High	+	‒	‒	Key enzyme in glyoxylate bypass, contributes to MTB persistence and virulence	No	glcB, ackA, fadB2, aceAb, aceAa
Rv1130 (prpD)	526	C, CW	Latent	Moderate	High	+	‒	NA	Metabolism of short-chain fatty acids	No	prpC, prpR, Rv1998c, Rv1132, icl1
Rv1169c (PE11, lipX)	100	E	NA	Low	Moderate	‒	‒	‒	Cell wall lipid remodeling and virulence	No	PPE17, Rv0774c, mshB, Rv0963c, lipF
Rv1793 (esxN)	94	E, M	NA	High	Moderate	+	‒	‒	Unknown	No	esxK, esxJ, esxP, esxA, esxB
Rv2629	374	C, CW, M	Latent	Moderate	High	+	‒	‒	Unknown	It performs well in preclinical trials and may be a good candidate vaccine	Rv2630, rpsC, rplW, rtcB, hemK
Rv3874 (esxB, CFP-10, lhp)	100	E, CW	Early	High	Low	+	‒	‒	Secreted protein involved in immune modulation and bacterial escape	AEC/BC02, GamTBvac, MVATG18598	esxA, eccCb1, esxH, PPE68, espC
Rv3619c (esxV)	94	E, CW	NA	High	Moderate	+	‒	‒	Possible virulence factor	ID93 + GLA-SE, ID91 repRNA	esxW, esxP, esxK, esxA, esxB
Rv3620c (esxW)	98	E	NA	Moderate	Low	+	‒	‒	Unknown	ID93 + GLA-SE	esxV, esxl, esxO, esxB, esxA
Rv3615c (espC, snm9)	103	E, CW	NA	High	Moderate	+	+	‒	ESX-1 secretion and virulence	H107e/CAF®10b, P846	espA, espD, esxB, esxA, espB
Rv0125 (pepA, mtb32a)	355	E, CW	NA	Very High	High	‒	+	‒	Immune response and proteolytic activity	M72/AS01E, MTB72F-AS02A	mpa, treS, PPE18, glpK, prcB
Rv1196 (PPE18, mtb39a)	391	CS, E, M	Latent	Moderate	Low	‒	‒	‒	Immune modulation and virulence	M72/AS01E, MTB72F-AS02A	esxK, esxL, PE13, esxW, esxV
Rv2608 (PPE42)	580	NA	NA	Moderate	High	‒	‒	NA	May play a role in inducing a humoral immune response	ID93 + GLA-SE	espE, PE_PGRS30, PE_PGRS16, PE_PGRS28, PE_PGRS15
Rv2930 (fadD26)	583	C, M	NA	Moderate	High	+	‒	NA	Involved in the activation and elongation of long-chain fatty acids	It performs well in preclinical trials and may be a good candidate vaccine	ppsA, ppsB, ppsE, ppsC, ppsD
Rv3876 (espI)	666	C, M	NA	Moderate	Moderate	+	‒	NA	Inhibits ESX-1-mediated secretion under low ATP conditions	H107e/CAF®10b	eccD1, espJ, esxA, espE, esxB
Rv3616c (espA)	392	M, E	NA	Low	High	‒	‒	‒	Required for the secretion of EsxA and EsxB, and involved in bacterial virulence	H107e/CAF®10b	espC, espD, esxB, esxA, Rv3612c
Rv2875 (mpt70)	193	EM, E	NA	High	Low	‒	+	‒	May be involved in immune modulation	H107e/CAF®10b	dipZ, sigK, nuoD, mpt64, rskA
Rv2873 (mpt83)	220	EM, M	NA	Moderate	Low	‒	‒	‒	Recognized by human and mouse T cells during MTB infection	H107e/CAF®10b	sigK, nuoD, dipZ, esxA, IpqH
Rv3478 (PPE60, mtb39c)	393	NA	NA	Low	Low	‒	‒	‒	Unknown	ID91 repRNA	PE31, Rv0178, Rv1944c, Rv0139, Rv3479
Rv3425 (PPE57)	176	CS, E, M	Latent	Low	High	+	+	‒	May play a role in the interaction with host immune system	It performs well in preclinical trials and may be a good candidate vaccine	PPE58, Rv0310c, Rv2659c, Rv3424c, PPE39
Rv0287 (esxG)	97	C, E, P	Early	Moderate	Moderate	‒	‒	‒	Part of the EsxGNAEsxH complex that disrupts host ESCRT function and facilitates bacterial growth	MVATG18598	esxH, espG3, esxR, eccD3, PPE4
Rv1737c (narK2, narK-3)	395	M	Latent	Low	High	‒	‒	‒	Involved in the transport of nitrate and nitrite	It performs well in preclinical trials and may be a good candidate vaccine	narX, narG, narl, narH, nirB
Rv3407 (vapB47)	99	NA	Latent	Low	High	+	‒	‒	Neutralizes the action of VapC47 toxin	MVATG18598、rBCGΔureC∷hly、rBCG AERAS-407	vapC47, fbpA, rpfB, phoY2, PPE42
Rv1818c (PE_PGRS33)	498	M, CS, E, CW	NA	Very High	Very High	‒	+	‒	Induces TNF-α release and macrophage apoptosis through TLR2 signaling pathway	It performs well in preclinical trials and may be a good candidate vaccine	PPE41, Rv0774c, PE10, PPE18, bacA
Rv0081	114	M	Latent	Low	Moderate	‒	‒	‒	NA	It performs well in preclinical trials and may be a good candidate vaccine	Rv0082, Rv0083, hycE, hycP, hycQ
Rv3812 (PE_PGRS62)	504	E	NA	Low	High	‒	‒	NA	Inhibits phagosome maturation and iNOS expression, supports mycobacterial survival	It performs well in preclinical trials and may be a good candidate vaccine	Rv2133c, alaS, Rv1972, helZ, Rv1691
Rv1735c	197	M	Latent	Moderate	High	‒	‒	‒	NA	It performs well in preclinical trials and may be a good candidate vaccine	ctpF, rip3, Rv1519, echA17, Rv2624c
Rv1736c (narX)	652	M, CW	Latent	Moderate	Very High	+	‒	NA	NA	It performs well in preclinical trials and may be a good candidate vaccine	narH, narK2, narl, narU, narK1
Rv0915c (PPE14)	423	NA	NA	High	High	‒	‒	‒	NA	It performs well in preclinical trials and may be a good candidate vaccine	PE7, Rv0890c, lipC, Rv0914c, Rv0913c
Rv1997 (ctpF)	905	CW, M	Latent	Moderate	Very High	‒	‒	NA	NA	It performs well in preclinical trials and may be a good candidate vaccine	rip3, acg, Rv2028c, pfkB, narK2
Rv1789 (PPE26)	393	NA	NA	Moderate	High	‒	‒	‒	NA	It performs well in preclinical trials and may be a good candidate vaccine	PE18, PPE25, Rv1147, adhA, Rv3095
Rv3018c (PPE46)	434	NA	NA	Moderate	High	‒	‒	‒	NA	It performs well in preclinical trials and may be a good candidate vaccine	PE27A, esxQ, esxR, eskS, Rv2738c
Rv2034	107	NA	NA	Low	High	+	‒	‒	Regulates lipid metabolism and hypoxic response, involved in stress response and lipid metabolism regulation	It performs well in preclinical trials and may be a good candidate vaccine	Rv2035, Rv2036, relF, Rv2640c, mbcA
Rv1039c (PPE15, mper1)	391	NA	NA	Moderate	High	‒	‒	‒	May play a key role in the homeostasis of triacylglycerol droplets in MTB and affect the pathogen’s entry into a dormant state	It performs well in preclinical trials and may be a good candidate vaccine	PE8, espG5, esxJ, esxl, Rv2633c
Rv3136 (PPE51)	380	M, CW	NA	Moderate	High	‒	‒	‒	Involved in the response to starvation and stress, may be part of the host environment within the host	It performs well in preclinical trials and may be a good candidate vaccine	PPE50, PE_PGRS20, hisN, ephD, Rv1747
Rv1172c (PE12)	308	NA	NA	Moderate	High	‒	‒	‒	Unknown	It performs well in preclinical trials and may be a good candidate vaccine	vapB28, fbiC, nuol, hsdM, ctaE
Rv0159c (PE3)	468	E	NA	Moderate	High	‒	‒	‒	Plays a role in the persistence of mycobacteria during infection and modulates the host immune response	It performs well in preclinical trials and may be a good candidate vaccine	PE4, Rv1364c
Rv3808c (glfT2, glfT)	637	C, M	NA	Moderate	High	+	‒	NA	Unknown	It performs well in preclinical trials and may be a good candidate vaccine	gIfT1, glf, ubiA, rfbD, Rv3807c
Rv2524c (fas)	3069	C, CW, M	NA	Moderate	Very High	+	‒	NA	Unknown	It performs well in preclinical trials and may be a good candidate vaccine	pks6, accD3, pks4, pks5, pks7
Rv0341 (iniB)	479	NA	NA	Very High	High	‒	‒	‒	Unknown	It performs well in preclinical trials and may be a good candidate vaccine	iniC, iniA, wbbL2, Rv3727, Rv2209
Rv2659c	375	NA	Latent	Moderate	High	+	‒	‒	Required for site-specific recombination to integrate the phage into the host genome	rBCGΔureC∷hly	Rv2658c, Rv2650c, Rv2656c, Rv2657c, Rv2655c
Rv2770c (PPE44)	382	NA	NA	Moderate	High	‒	‒	‒	Potential antigen candidate for a subunit vaccine against TB with protective efficacy comparable to BCG	It performs well in preclinical trials and may be a good candidate vaccine	PE8, espG5, Rv2336, csm5, Rv1320c
Rv1984c (cut7)	217	E, CW	NA	Moderate	Moderate	‒	+	‒	Esterase activity, involved in the induction of delayed-type hypersensitivity and cytokine release	It performs well in preclinical trials and may be a good candidate vaccine	Rv3802c, mpt64, Rv0045c, esxB, Rv1592c
Rv3803c (mpt51, fbpC1, fbpD, mpb51)	299	E	NA	Moderate	High	+	‒	‒	May play a role in host tissue attachment and immune modulation	It performs well in preclinical trials and may be a good candidate vaccine	Rv3802c, hspX, esxA, mpt63, fbpA
Rv1195 (PE13)	99	E	NA	Moderate	Moderate	‒	+	‒	May be involved in MTB-host interactions and contribute to the pathogen’s survival within macrophages	It performs well in preclinical trials and may be a good candidate vaccine	PPE18, esxL, esxK, PE_PGRS11, Rv0485
Rv2660c	75	NA	Latent	Very High	Moderate	‒	+	‒	Unknown, potential T-cell antigen for vaccine development	H56:IC31, p846	Rv2661c, Rv2558, usfY, Rv0061c, Rv1954c

^a^*Abbreviations*: *C* Cytoplasm, *CM* Cytoplasmic membrane, *CS* Cell surface, *CW* Cell wall, *E* Extracellular space, *EM* Extracellular matrix, *ER* Extracellular region, *P* Phagosome, *M* Membrane

^b^Antigenicity: The antigenicity of proteins is evaluated using the VaxiJen v2.0 server, with a threshold value set at 0.4. In this study, proteins are categorized into four levels of antigenicity based on their antigenicity scores: scores below 0.4 are classified as Low, scores between 0.4 and 0.6 are Moderate, scores between 0.6 and 0.8 are High, and scores of 0.8 or above are Very High

^c^Immunogenicity: Scores below 0 are classified as Low, scores between 0 and 1 are Moderate, scores between 1 and 10 are High, and scores of 10 or above are Very High

^d^Hydrophilicity:

^+^Indicates that the antigen has high hydrophilicity, meaning it has good solubility in aqueous solutions or strong interactions with water molecules

^‒^Indicates that the antigen has low hydrophilicity, meaning it has poor solubility in aqueous solutions or weak interactions with water molecules

^e^Allergenicity:

^+^Indicates that the antigen may have allergenic properties, meaning it has the potential to trigger allergic reactions in the immune system

^‒^Indicates that the antigen has no known or discovered allergenic properties, meaning it is unlikely to cause allergic reactions

^f^Toxicity:

^+^Indicates that the antigen has toxicity, potentially causing harm or having harmful effects on organisms

^‒^Indicates that the antigen has no toxicity, or no toxicity has been discovered yet

*NA* Not Available or Not Applicable

### Role of bioinformatics and AI in antigen selection

The integration of bioinformatics and AI has revolutionized the process of antigen selection in the development of TB vaccines [41]. These technologies enable the systematic analysis and interpretation of vast amounts of biological data, accelerating the identification of potential antigens with high precision and efficiency [42].

Bioinformatics is essential for managing and analyzing the genomic and proteomic data of MTB. By using databases and computational tools, researchers can predict antigenic epitopes, understand genetic variations across different strains, and model the structural interactions between antigens and the host immune system. This computational approach allows for the efficient narrowing down of potential antigens for further experimental validation, thereby saving both time and resources. In our previous studies, we have developed several multi-epitope vaccines (MEVs) using bioinformatic tools [20, 43–45]. For instance, we utilized the IEDB database for screening immunodominant T cell epitopes, the ABCpred server for predicting B cell epitopes, and the ANTIGENpro and VaxiJen v2.0 servers for predicting biological properties. Additionally, we employed Allergen FP v.1.0 and AllerTOP v.2.0 for allergenicity prediction, the Expasy ProtParam server for determining physicochemical parameters, the protein-sol server for assessing solubility, and PSIPRED for evaluating the secondary structure of the multi-epitope vaccine. The integrated use of these tools provides a scientific basis for the design and optimization of TB vaccine candidate antigens.

Furthermore, AI enhances these efforts by applying machine learning algorithms to identify patterns and features associated with effective immune responses. The application of AI technology, particularly AlphaFold 2 and its deep learning capabilities, has revolutionized protein structure prediction by achieving atomic-level accuracy [46]. Several studies have utilized AlphaFold 2 in vaccine design, significantly enhancing both the accuracy and speed of the development process [47–49]. The introduction of AlphaFold 3 has further advanced predictive accuracy by enabling the prediction of complex structures of virtually all molecule types without requiring structural information input [50]. In the context of vaccine design, understanding the spatial structure of antigens is essential for molecular docking and dynamic simulations. Tools such as PatchDock, AutoDock Vina, GROMACS, and AmberTools are employed to predict how antigens bind to immune receptors and to simulate the dynamic behavior of these complexes [51–54].

In the next section, to predict the immunogenicity and antigenicity of the key TB antigens under investigation, we utilized VaxiJen v2.0 and IEDB Immunogenicity server for evaluation. Additionally, the Expasy ProtParam tool was used to assess the aliphatic index and grand average of hydropathicity of the antigens, while AllerTOP v.2.0 and ToxinPred were employed to predict antigen allergenicity and toxicity [55, 56]. We focused particularly on 12 MTB antigens with high candidate potential. High-precision protein structure predictions for these antigens were performed using the AlphaFold server, and their protein interactions were predicted using the STRING database [55–59]. Furthermore, immune simulation predictions conducted through the C-ImmSim server analyzed the ability of these antigens to induce antibodies and cytokines, providing valuable information for vaccine design and antigen selection [60–63].

### Key TB antigens under investigation

#### EsxA and EsxB

**Fig. 1:**
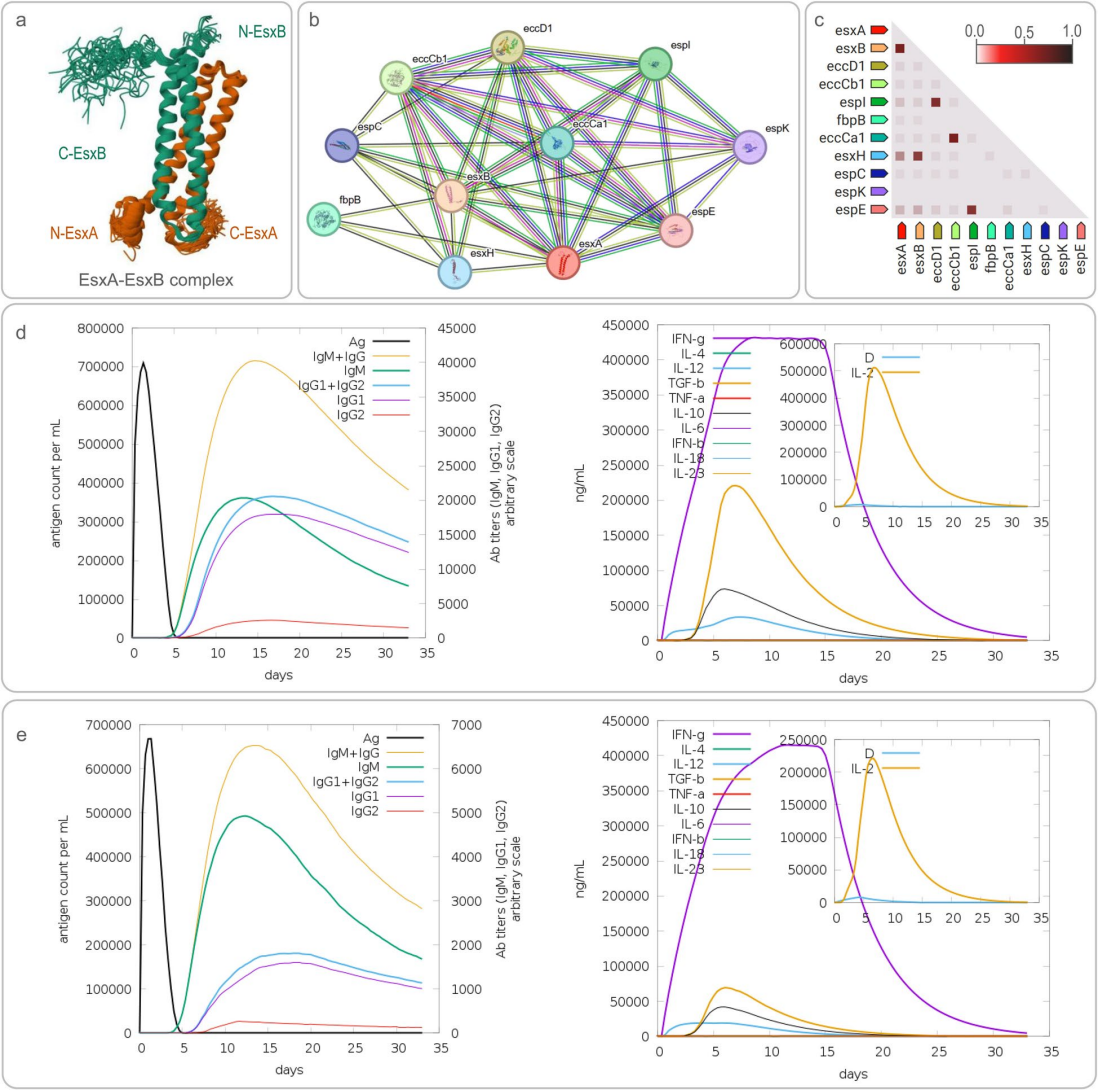
EsxA-EsxB Complex: Structural Insights, Interactions, and Immunological Implications in *Mycobacterium tuberculosis*. (**a**) Solution Structure of the EsxA-EsxB Complex. PDB https://doi.org/10.2210/pdb1WA8/pdb; (**b**) Proteins interacting with EsxA and EsxB; (**c**) Observed coexpression in *Mycobacterium tuberculosis* H37Rv. In the triangle-matrices above, the intensity of color indicates the level of confidence that two proteins are functionally associated, given the overall expression data in the organism; (**d**, **e**) Predicted IgM and IgG antibodies and cytokines production induced by EsxA and EsxB

EsxA (ESAT-6, Rv3875) and EsxB (CFP-10, lhp, mtsA10, Rv3874) are two closely related proteins encoded by the RD-1 region of MTB, playing crucial roles in the pathogenesis, diagnosis, and vaccine development for TB [29, 64–67]. These two proteins are secreted through the ESAT-6 secretion system 1 (ESX-1), also known as the type VII secretion system (T7S) [68]. Notably, the RD-1 region is present in pathogenic *M. bovis* and MTB but absent in BCG strains [69]. EsxA and EsxB form a stable 1:1 complex [70, 71] (Fig. 1a) and interact with other proteins such as eccD1, eccCb1, espl, fbp, esxH, PPE68, and espC (Fig. 1b, c). This complex plays a significant role in MTB virulence. Alexander S. Pym et al. observed that reintroducing ESAT-6 into BCG resulted in significant changes in colony morphology and enhanced virulence [72].

Regarding immune responses, both EsxA and EsxB are highly immunogenic, recognized by over 70% of TB patients [73], making them valuable for TB diagnosis in humans and animals [74, 75]. These proteins not only induce B cell-mediated humoral immunity but also activate T lymphocytes to produce IFN-γ (Fig. 1d and e) [76–79]. Studies have shown that using a mixture of EsxA and EsxB, and TB7.7 as specific antigens can induce MTB-specific immune responses through whole blood stimulation, producing cytokines and chemokines [80]. Antony M Rapulana et al. further discovered that joint stimulation of whole blood with EsxA and EsxB could use IFN-γ, IL-2, and IP-10 as potential biomarkers to distinguish between LTBI and healthy individuals [81].

Moreover, EsxA and EsxB play important roles in MTB virulence [72], leading to host cell lysis [66] and suppression of macrophage responses [82]. Studies have shown that pretreatment of macrophages with recombinant EsxB protein can reduce the production of reactive oxygen and nitrogen species [65, 83, 84]. Notably, EsxB can induce potent cytotoxic T lymphocyte (CTL) responses, accounting for up to 30% of CD8 + T cells in the lungs of MTB-infected mouse models [85]. This finding provides new hope for developing vaccine strategies aimed at inducing CD8 + T cell responses [29]. Given the widespread application of EsxA and EsxB in immunological research and vaccine design, several vaccines, including AEC/BC02, GamTBvac, H56:IC31, and MVATG18598, have incorporated these two proteins as key antigens [86–90].

#### TB10.4 (esxH, cfp7, Rv0288)

**Fig. 2:**
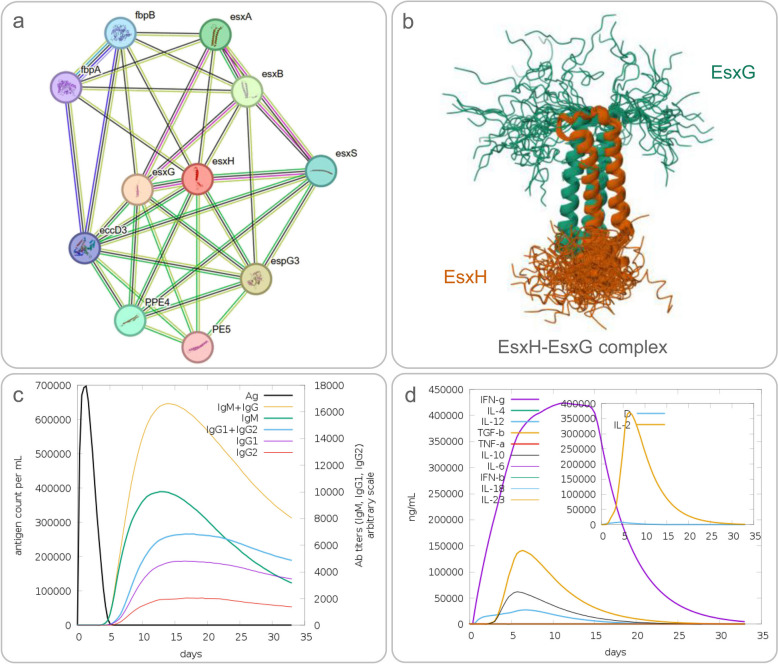
EsxH Protein Interactions and Immunological Responses: Structural and Predictive Analysis. (**a)** Proteins interacting with EsxH; (**b**) Solution structure of the EsxA-EsxB Complex, PDB https://doi.org/10.2210/pdb2KG7/pdb; (**c**) Predicted IgM and IgG antibody response induced by EsxH; (**d**) Predicted cytokine response induced by EsxH

TB10.4, also known as esxH, cfp7, or Rv0288, is a secretory antigen protein isolated from the serum of cattle infected with *Mycobacterium bovis* [91]. As a member of the ESAT-6 protein family, TB10.4 interacts with proteins such as esxG, espG3, esxB, esxA, and PPE4 (Fig. 2a), which are crucial for its biological functions. EsxG and EsxH have previously been shown to form a stable 1:1 heterodimeric complex, characterized by a core four-helix bundle decorated at both ends by long, highly flexible N- and C-terminal arms, which contain numerous highly conserved residues (Fig. 2b) [92]. Encoded by the Rv0288 gene, TB10.4 is downregulated in the attenuated H37Ra strain and plays a crucial role in inducing CD4 + T cells and cytotoxic T lymphocytes (CTLs), which are vital for controlling intracellular pathogens like MTB (Fig. 2c) [91, 93]. It also activates B-cells, leading to the production of antibodies that can neutralize pathogens and provide immune protection (Fig. 2d). This highlights its central role in mycobacterial pathogenesis and T cell activation [94].

With respect to the viral vector vaccine strategy, a study by Mariia Sergeeva and colleagues utilized an attenuated influenza virus vector vaccine Flu/THSP expressing MTB antigens TB10.4 and HspX to immunize mice and guinea pigs via the intranasal route [95]. This approach successfully induced CD4^+^ and CD8^+^ T cell immune responses and promoted the production of cytokines such as IFN-γ, IL-2, and TNF-α. These immune responses reduced bacterial load in mouse lungs and spleen and improved lung pathology. Notably, compared to the use of BCG alone, the priming with BCG followed by boosting with Flu/THSP vaccine significantly reduced the bacterial load in the lungs and spleens of guinea pigs and markedly improved the pathological morphology of their lungs [95].

Furthermore, TB10.4 has shown significant immunogenicity and is strongly recognized by T cells from TB patients and BCG vaccinees. Compared to ESAT-6, TB10.4 is more strongly immunologically recognized in TB patients [96]. Building on the Ag85B-ESAT-6 fusion protein, Jes Dietrich and colleagues replaced ESAT-6 with TB10.4 to evaluate its potential as a TB subunit vaccine. Results showed that the Ag85B-TB10.4 fusion protein was comparable in immunogenicity to the BCG vaccine, and TB10.4 was more strongly recognized than ESAT-6 [97]. This suggests that TB10.4 has the potential to be an ideal substitute for ESAT-6, providing a new perspective for TB vaccine development. Currently, TB10.4 has become one of the antigen components in TB vaccines such as TB/FLU-05E and MVATG18598, and its application prospects in vaccine development are promising.

#### esxV (Rv3619c) and esxW (Rv3620c)

**Fig. 3:**
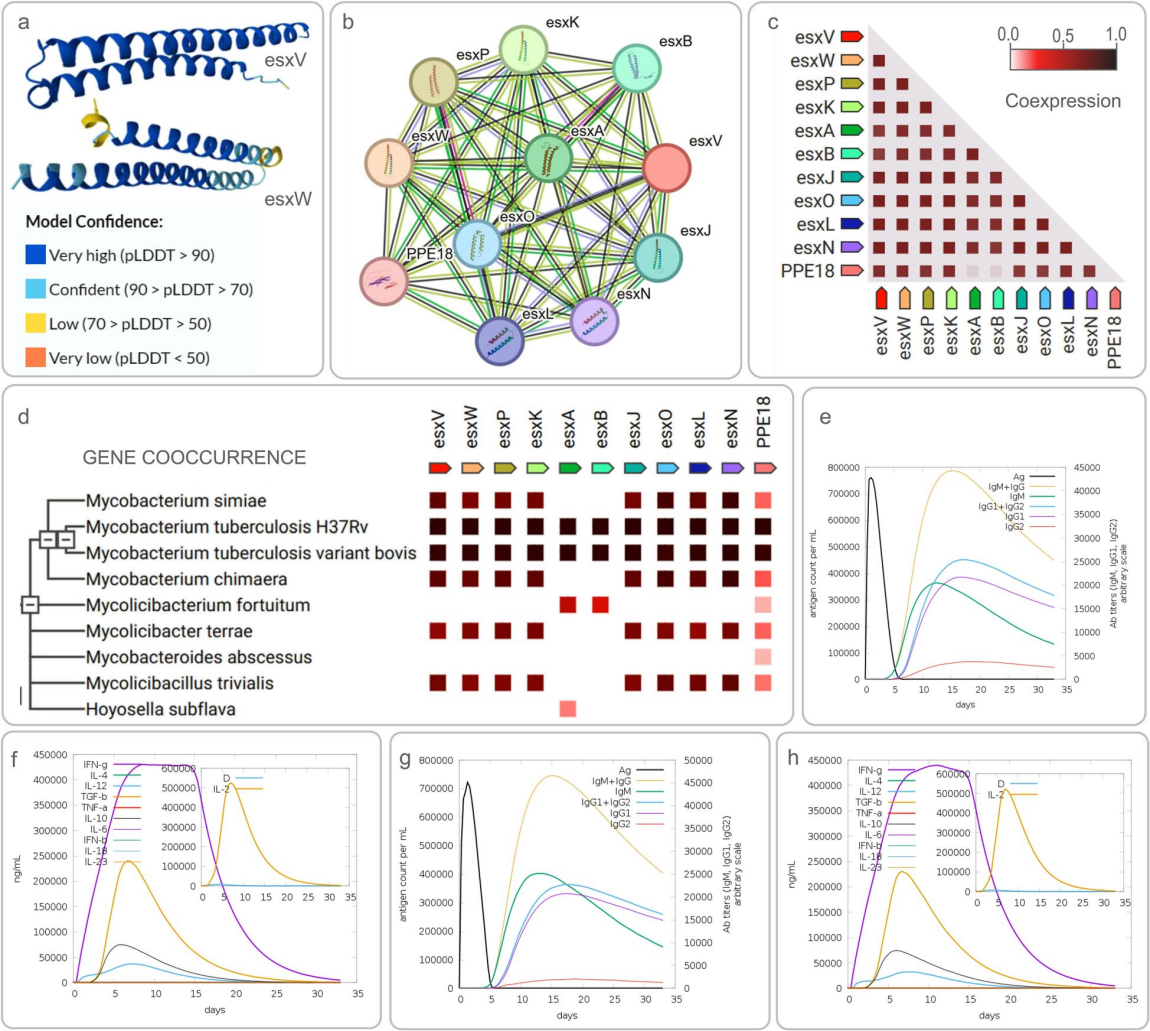
Structural and Functional Profiling of EsxV and EsxW in Mycobacterium tuberculosis: Prediction of Immunological Outcomes. (**a**) 3D structure of esxV and esxW predicted by AlphaFold. The 3D spatial structure prediction of the both proteins was done by AlphaFold, and the accuracy of the prediction is expressed by per-residue confidence score (pLDDT) (0–100), with higher scores representing higher accuracy, and the graph indicates a gradual increase in prediction accuracy from orange to blue; (**b**) Proteins interacting with esxV and esxW; (**c**) Observed coexpression in *Mycobacterium tuberculosis* H37Rv; (**d**) Gene cooccurrence. Gene families whose occurrence patterns across genomes show similarities; **e** Predicted IgM and IgG antibody response induced by esxV; (**f**) Predicted cytokine response induced by esxV; (**g**) Predicted IgM and IgG antibody response induced by esxW; (**h**) Predicted cytokine response induced by esxW

EsxV and EsxW are two immunodominant antigens specific to MTB, composed predominantly of alpha-helices and belonging to the ESAT-6 family (Fig. 3a). These antigens are known to interact with a variety of proteins, including EsxL, EsxO, EsxP, EsxK, EsxA, and EsxB (Fig. 3b, c) [98]. These interactions are crucial for their biological functions. esxW can stimulate Th1 immune responses and increase IFN-γ production in peripheral blood mononuclear cell (PBMCs) from healthy tuberculin-positive (PPD +) donors [99]. The genes encoding esxV and esxW are both located in the RD-9 of the MTB genome (Fig. 3d) [64]. Similar to ESAT-6/CFP10, esxV and esxW can form a 1:1 heterodimer complex through interaction [100]. Research indicates that the choice of adjuvants and delivery systems significantly influences the cytokine responses, including those of Th1, Th2, Th17, and Treg, in mice immunized with MTB antigens. Notably, EsxV is the sole antigen that consistently induces antigen-specific Th1 responses and IFN-γ secretion across all tested adjuvants and delivery systems. When combined with alum or Mycobacterium smegmatis, EsxV also induces significant levels of antigen-specific IgG antibodies (Fig. 3e, f) [101]. EsxW, similarly, induces protective immune responses, promoting IFN-γ production and T cell activation, which suggests its potential as a component in MTB vaccine development (Fig. 3g, h). Based on these findings, esxV and esxW have been incorporated as components in the ID93 + GLA-SA vaccine, while the ID91 repRNA vaccine has also selected esxV as one of its antigens, further demonstrating their potential in TB vaccine development.

#### Ag85A (fbpA, mpt44, Rv3804c)

Ag85 complex, also known as Fibronectin-binding proteins (Fbps), is a 30–32 kDa family consisting of three proteins: Ag85A, Ag85B, and Ag85C. These proteins are located in the MTB cell wall, have secretory capabilities [102], and possess important enzymatic mycobacterial acyltransferase activity [103]. As one of the most abundantly secreted proteins by MTB [104], the Ag85 complex can elicit strong humoral and cellular immune responses [105–107], making it a promising candidate antigen for TB vaccine development. Ag85A, a diacylglycerol acyltransferase involved in lipid body formation (Figure S1a) [108], interacts with various proteins such as fbpC, fbpB, lipY, dagK, and esxA (Figure S1b), demonstrating potential to induce Th1-type cytokines [109]. It promotes the production of cytokines including IFN-γ, TGF-β, IL-10, and IL-12 (Figure S1c), while also activating B cells to produce Ag85A-specific antibodies (Figure S1d), exhibiting good immunogenicity [110, 111]. Ag85A and Ag85B extracted from BCG and MTB culture filtrates have successfully induced strong T cell proliferation and IFN-γ production in most LTBI patients, BCG-vaccinated mice, and humans. However, similar effects were not observed in TB patients [112, 113]. In a study by Marta Romano et al., mice initially immunized with an Ag85A-encoding DNA plasmid followed by BCG boost showed significantly improved survival rates and enhanced CD4^+^ and CD8^+^ T cell-mediated IFN-γ responses [114]. Conversely, a regimen involving initial BCG immunization followed by an Ag85A-encoding DNA plasmid boost promoted IFN-γ secretion and IL-17 production but did not improve mouse survival rates. Based on these findings, Ag85A has been selected as a primary candidate antigen for MTB vaccine development. Several vaccines, including GamTBvac, Ad5Ag85A, TB/FLU-01L, TB/FLU-04L, ChAdOx1.85A, and MVA85A, have incorporated Ag85A into their antigen components.

#### Ag85B (fbpB, mpt59, Rv1886c)

Ag85B, a key secretory protein of MTB, plays multiple biological roles. It not only participates in cell wall mycolyltransferase activity, promoting cell wall synthesis (Figure S1e) [115, 116], but is also considered an important target due to its potential in immune protection and drug development. Ag85B interacts with proteins such as fbpC, Rv1885c, fbpA, esxA, and mpt64 (Figure S1f), inducing B cell-mediated humoral immune responses (Figure S1g) and eliciting T cell responses in humans and mice, particularly enhancing the production of the Th1-type cytokine IFN-γ (Figure S1h) [117]. In animal model studies, an immunization strategy combining the mincle agonist UM-1098 and ESAT6/Ag85B successfully induced protective Th1 and Th17 immune responses, significantly increasing the secretion levels of IFN-γ, IL-17A, tumor necrosis factor-α (TNF-α), IL-5, and other cytokines, as well as antigen-specific IgG, IgG2c, and IgG1 antibodies, while reducing bacterial load in mouse lungs [118]. A. Weinrich Olsen and colleagues developed a TB subunit vaccine based on the Ag85B and ESAT-6 fusion protein, which induced effective long-term memory immunity in mice, providing protection levels comparable to the BCG vaccine and demonstrating high efficacy against TB [119]. Research by Marcus A. Horwitz et al. also confirmed that rBCG30 expressing Ag85B, used as a booster for the BCG vaccine, significantly enhanced BCG's protective efficacy [120]. Given Ag85B's significant role in eliciting immune responses, it has become a popular candidate antigen in vaccine development. Currently, several vaccines, including AEC/BC02, H56:IC31, MVATG18598, rBCG30, and ID91 repRNA, incorporate Ag85B as a component.

#### PPE18 (mtb39a, Rv1196)

PPE18, also known as MTB39a or Rv1196, is a cell wall-associated protein encoded by the Rv1196 gene (Figure S2a) [121]. As a member of the PE/PPE protein family, PPE18 interacts with various proteins such as esxK, esxL, PE13, esxW, and esxV (Figure S2b). This family accounts for a significant proportion (8–10%) of the MTB genome and is characterized by the Pro-Pro-Glu (PPE) repeat sequence in the N-terminal region [122]. PPE family proteins have the ability to regulate cytokine secretion and influence host cell apoptosis and necrosis [123–126]. PPE18 exhibits significant immunogenicity (Figure S2c and d) and can bind to Toll-like receptor 2 (TLR2) on the surface of macrophages. Through activation of the p38 mitogen-activated protein kinase (MAPK) signaling pathway, it promotes the production of the anti-inflammatory cytokine IL-10, thereby promoting a Th2-type immune response [127]. Simultaneously, PPE18 suppresses the Th1-type immune response by specifically downregulating pro-inflammatory cytokines such as IL-12 and TNF-α. Additionally, PPE18 can inhibit antigen presentation by MHC class II molecules, impair CD4^+^ T cell activation, and suppress B cell activation and antibody production [128]. These characteristics reveal PPE18's crucial role in MTB immune evasion [29].

The virulence factor properties of PPE18 have also been confirmed in mouse models. Infection with MTB strains lacking PPE18 resulted in reduced bacterial load, less organ damage, and increased survival rates in mice [129]. Furthermore, a DNA vaccine encoding PPE18 showed protective effects in mice, although not as effective as the BCG vaccine, but significantly reduced lung bacterial burden compared to the control group [130]. These findings support further research into PPE18 as a vaccine candidate component. Currently, some vaccines such as M72/AS01E have incorporated PPE18 into their antigen combinations.

#### Rv1813c

Rv1813c is a latency antigen discovered in MTB (Figure S2e) [131], co-regulated by regulatory factors MprA and DosR. As a conserved hypothetical protein, Rv1813c interacts with various proteins such as Rv2030c, acg, Rv3127, Rv1733c, and fdxA (Figure S2f), which are crucial for its functions. A key function of Rv1813c is its ability to target host cell mitochondria and manipulate host metabolic pathways [132]. When expressed in eukaryotic cells, Rv1813c localizes to the mitochondrial intermembrane space and increases ATP production in host cells by promoting the oxidative phosphorylation pathway. This metabolic adjustment is beneficial for MTB as it helps avoid the generation of reactive oxygen species (ROS) and cell apoptosis. Moreover, cells expressing Rv1813c show delayed release of cytochrome c from mitochondria, which is part of the early defense mechanism of macrophages infected with MTB. Thus, Rv1813c influences both the metabolic and apoptotic responses of host cells [132].

Rv1813c also demonstrates strong immunogenicity (Figure S2g and h) and can be recognized by over 70% of TB patients [133]. In mouse models, Rv1813c can induce strong CD4^+^ T cell responses, achieved through the expression of IFN-γ/TNF and TNF/IL-2 cytokines. Compared to other candidate antigens such as Rv3620, Rv3619, Rv2608, and Rv3478, Rv1813c induces the highest number of CD8 + T cells expressing IFN-γ and/or TNF [99]. Furthermore, Rv1813c can provide protective effects in MTB-infected mice by generating antigen-specific IgG2c antibodies to reduce bacterial load in the lungs. Given these characteristics of Rv1813c, it has been incorporated into subunit vaccines ID93 + GLA-SE and MVATG18598 as a potential vaccine component, aiming to enhance protection against TB.

#### pepA (mtb32a, Rv0125)

**Fig. 4:**
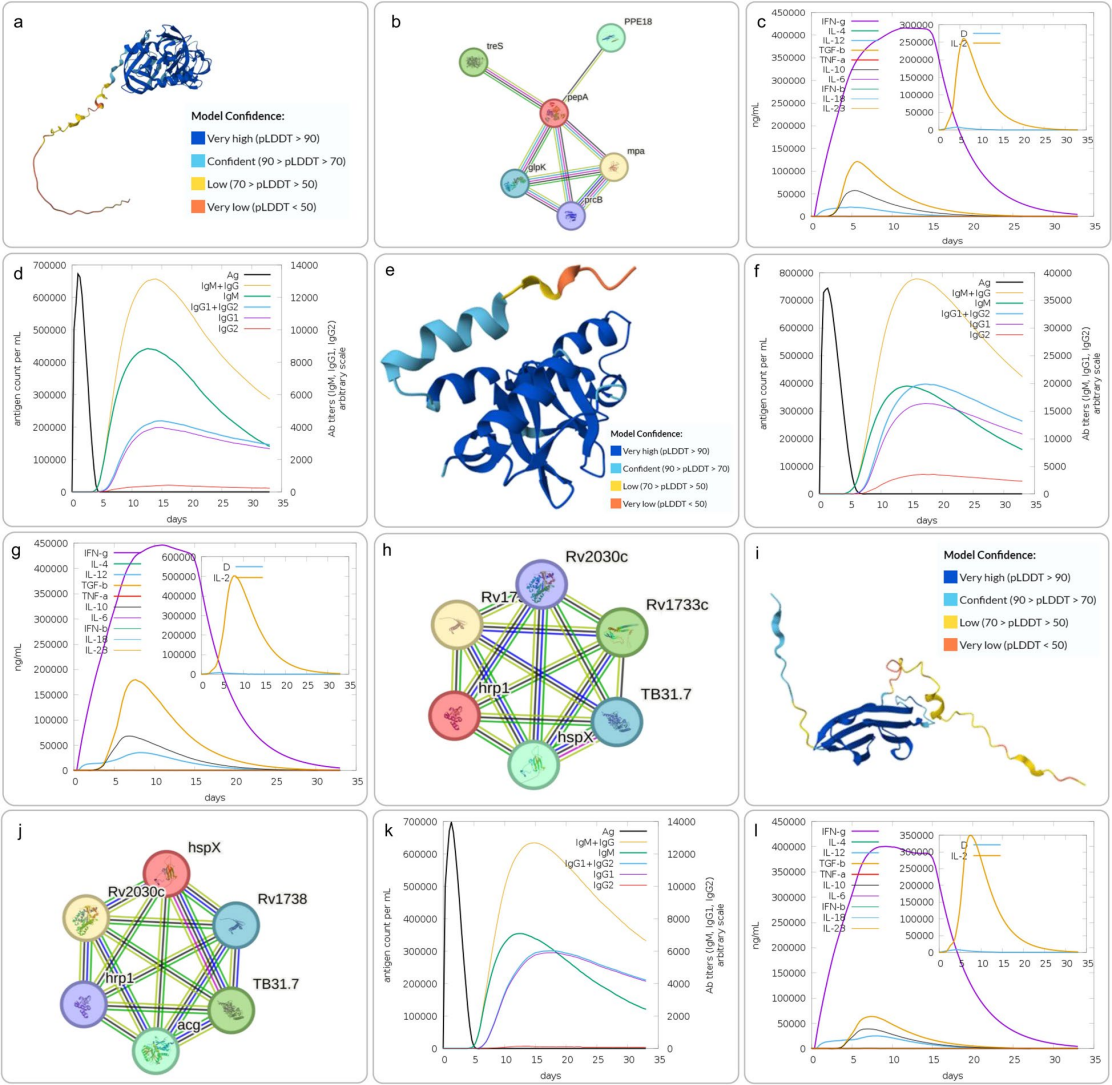
An overview of the computational predictions for pepA, Rv2626c, and Rv2031c. (**a**) 3D structure of pepA predicted by AlphaFold; (**b**) Proteins interacting with pepA; (**c**) Predicted cytokine response induced by pepA; (**d**) Predicted levels of antigen-special antibodies induced by pepA; (**e**, **i**) 3D structure of Rv2626c or Rv2031c predicted by AlphaFold; (**f**, **j**) Predicted levels of antigen-special antibodies induced by Rv2626c or Rv2031c; (**g**, **k**) Predicted cytokine response induced by Rv2626c or Rv2031c; (**h**, **l**) Proteins interacting with Rv2626c or Rv2031c

PepA, identified as a potential serine protease (Fig. 4a), has a function that is not yet fully understood but is generally considered to be a secretory protein [29]. This protein is encoded by a single-copy gene in the MTB complex, present in both pathogenic and non-pathogenic strains, including BCG strains [134]. PepA interacts with various proteins such as mpa, treS, PPE18, glpk, and prcB (Fig. 4b), and these interactions may be crucial for PepA's function. PepA demonstrates the ability to promote PBMC proliferation and can induce IFN-γ secretion (Fig. 4c) [134]. Additionally, PepA can induce B cell-mediated humoral immune responses (Fig. 4d). In a rhesus monkey model, during the first 2–3 months of early MTB infection, the levels of PepA-specific IgG antibodies in plasma were significantly higher in the LTBI group compared to the active tuberculosis (ATB) group. Moreover, the secretion levels of PepA-specific IgG antibodies in plasma increased before and during early infection in the LTBI group, suggesting that mucosal and systemic antigen-specific antibody responses might be associated with protection against TB [135]. Given the high conservation of PepA in mycobacteria [136], it is considered a promising vaccine target [137]. Based on these characteristics, PepA has been selected as one of the antigen components in vaccines such as M72/AS01E, providing new directions for developing novel TB vaccines.

#### Rv2626c (hrp1)

Rv2626c, also known as hrp1, is a hypoxia-responsive protein encoded by the open reading frame Rv2626c in MTB (Fig. 4e) [138]. Rv2626c expression is upregulated when MTB faces stressful conditions such as hypoxia or nitric oxide (NO) exposure, and it can be detected in both MTB culture supernatants and lysates. This protein not only plays a crucial role in MTB's stress response but also demonstrates significant immunogenicity, capable of eliciting specific antibodies and cytokines from the host (Fig. 4f and g). Rv2626c interacts with various proteins such as Rv1738, Rv1733c, hspX, TB31.7, and Rv2030c (Fig. 4h), and these interactions may have important implications for MTB's pathogenesis and immune evasion.

Studies have shown that recombinant Rv2626c protein (rRv2626c) can specifically bind to the surface of mouse macrophages, activating the NF-κB signaling pathway, thereby promoting NO production and inducible nitric oxide synthase (iNOS) expression [139]. Furthermore, rRv2626c can enhance the secretion of Th1-type cytokines IL-12, TNF-α, and IFN-γ in PBMCs from ATB patients. Rv2626c's impact on macrophage signaling also includes promoting the expression of co-stimulatory molecules B7-1, B7-2, and CD-40 on macrophage surfaces, which in turn promotes T cell proliferation [139]. These findings suggest that Rv2626c could be an effective T cell vaccine candidate antigen. Further research has shown that DNA vaccines encoding Rv2626c exhibit strong immunogenicity in both BALB/c and C57BL/6 mice, inducing high levels of IFN-γ, IL-2, and antigen-specific IgG antibodies [140]. In BALB/c mice, the IFN-γ levels induced by Rv2626c were comparable to those induced by Rv3804c encoding Ag85A [140]. These results further confirm the potential of Rv2626c as a latency-associated antigen. Given these characteristics of Rv2626c, it has been incorporated as a component in vaccines such as MVATG1859, demonstrating its promising prospects as a candidate antigen for TB vaccines.

#### Rv2031c (HspX, Hsp16.3, acr)

Rv2031c, commonly known as Heat Shock Protein X (HspX), is an α-crystallin protein encoded by the acr gene and is one of the downstream genes of the DosR regulatory system (Fig. 4i) [141]. Under hypoxic conditions, the expression levels of dormancy-associated genes, including Rv2031c, increase, which helps thicken the MTB cell wall and maintain its dormant state, allowing MTB to survive long-term in the host while avoiding clearance. Rv2031c interacts with various proteins such as Rv2030c, TB31.7, acg, Rv1738, and hrp1 (Fig. 4j). These interactions can induce specific humoral and cellular immune responses (Fig. 4k and l), providing protective effects against MTB infection.

Studies have shown that DNA vaccines containing Rv2031c exhibit immunogenicity in mouse models and demonstrate protective effects in guinea pig models [142]. Rv2031c can induce the secretion of IFN-γ and IL-10 in mice, as well as high levels of IgG2a and IgG1 antibodies. In guinea pig models, Rv2031c reduced bacterial load in the spleen and lungs and decreased the number of granulomas in lung tissue [142]. Furthermore, immunization of BALB/c and C57BL/6 mice with DNA plasmids encoding Rv2031c can induce high levels of IFN-γ, IL-2, and antigen-specific IgG antibodies. In BALB/c mice, the IFN-γ levels induced by Rv2031c were higher than in C57BL/6 mice [140]. Notably, Rv2031c protein can be detected in exosomes isolated from the plasma of TB patients but not in healthy individuals, suggesting that Rv2031c has potential as a biomarker for TB diagnosis [143]. Given these characteristics of Rv2031c, it is considered a promising candidate antigen for preventive vaccines. Currently, the TB/FLU-05E viral vector vaccine has incorporated Rv2031c as one of its antigens, further confirming the importance of Rv2031c in TB vaccine development.

## Promising TB vaccines in preclinical and clinical studies

### Preclinical vaccine candidates

Preclinical research is a pivotal phase in the vaccine development process. It involves assessing the safety, efficacy, and immunogenicity of vaccines in animal models. This phase is crucial for providing data support for subsequent clinical trials, reducing risks, and optimizing vaccine preparation processes. Therefore, it is essential to pay attention to the promising tuberculosis vaccines in preclinical research.

#### Attenuated live vaccines

Attenuated live vaccines represent a promising avenue in TB vaccine development due to their ability to mimic natural infection and induce robust immune responses. One such candidate is MTBΔsigH, an attenuated strain of MTB with a deletion in the sigH gene, which encodes a sigma factor involved in stress response [144]. Studies by Deepak Kaushal and colleagues have demonstrated that MTBΔsigH, when administered via the aerosol route in rhesus macaques, effectively induces CD4^+^ and CD8^+^ T cell responses and a robust inducible bronchus-associated lymphoid tissue (iBALT) reaction. Notably, MTBΔsigH vaccination resulted in a significant reduction in bacterial load and pathological changes in the lungs compared to both the negative control and the BCG vaccinated groups, suggesting a high level of protective efficacy [145]. These findings underscore the potential of MTBΔsigH as a foundation for further development of tuberculosis vaccine candidates.

Another attenuated live vaccine candidate is MTBΔlpqS, a MTB mutant strain with a deletion in the lpqS gene [146]. In a guinea pig model infected with MTB, MTBΔlpqS was found to induce IFN-γ secretion while reducing IL-10 levels, which are associated with MTB immune evasion. Importantly, MTBΔlpqS provided superior protection compared to BCG, as evidenced by reduced bacterial counts in the lungs and spleens, and mitigated lung lesions. Interestingly, MTBΔlpqS also demonstrated protective effects in the liver and spleens of guinea pigs, with no observed lesions [146].

*M. paragordonae* (Mpg), a temperature-sensitive mycobacterium, has also shown promise as a live vaccine candidate. In a mouse model, Mpg was found to induce the maturation of bone marrow-derived dendritic cells (BMDC) and stimulate a Th1-type immune response, characterized by the secretion of IFN-γ, TNF-α, IL-2, and IL-12, as well as cytotoxic T lymphocyte (CTL) responses and IgG2a antibody production. Moreover, Mpg vaccination led to a reduction in bacterial counts in the lungs, liver, and spleens, and alleviated pulmonary inflammation [147]. These results position Mpg as a potential candidate for further investigation as a tuberculosis vaccine.

#### Viral vector vaccines

Viral vector vaccines have emerged as a powerful platform for TB vaccine development, leveraging the ability of viral vectors to deliver antigens and induce potent immune responses. One such candidate is LV::li-HAEPA, a lentiviral vector vaccine encoding a tandem sequence of EsxH, EsxA, EspC, and PE19 antigens, with an N-terminal addition of the MHC-II invariant chain (li) [148]. Jodie Lopez and colleagues reported that LV::li-HAEPA vaccination in mice induced antigen-specific CD4 + and CD8 + T cells producing IFN-γ and TNF. In comparison to BCG alone, a priming-boosting regimen with LV::li-HAEPA followed by LV::li-HAEPA plus cGAMP adjuvant resulted in significantly reduced bacterial loads in the lungs and spleens, demonstrating enhanced protective efficacy [148].

Another viral vector vaccine candidate is SeV85AB, a recombinant Sendai virus (SeV) vector encoding MTB antigens Ag85A and Ag85B [149]. In a mouse model, SeV85AB vaccination induced antigen-specific CD4^+^ and CD8^+^ T cells and high levels of lung-resident memory CD8^+^ T (TRM) cells. When compared to BCG or SeV85AB alone, a BCG priming-SeV85AB boosting regimen showed significantly improved protection, as evidenced by reduced bacterial loads and mitigated pathological changes in the lungs and spleens of mice [149].

SeV986A is a recombinant SeV vector vaccine expressing MTB antigens Rv2029c, Rv2028c, Rv3126c, and Ag85A [150]. In a mouse model, a BCG priming-SeV986A boosting regimen not only significantly enhanced antigen-specific CD4^+^ and CD8^+^ T cell responses but also provided stronger protection against MTB infection, reducing bacterial loads and pathological changes in the lungs. In a latent infection mouse model, compared to the PBS control group, the BCG priming-SeV986A boosting regimen significantly reduced bacterial loads in the lungs and spleens and decreased pulmonary inflammatory infiltration [150]. These findings highlight the potential of SeV986A as a vaccine candidate with significant protective effects in both acute and latent infection settings.

#### Recombinant BCG vaccines

Recombinant BCG vaccines offer a promising approach to enhancing the protective efficacy of the existing BCG vaccine. rBCG::XB is one such candidate, engineered to overexpress the Ag85B and HspX antigens [151]. In a mouse model, rBCG::XB significantly stimulated the production of Th1-type cytokines and antigen-specific IgG1 and IgG2a antibodies. It induced higher numbers of antigen-specific TNF-α + IFN-γ + , IFN-γ + , and TNF-α + CD4 + T cells compared to the BCG group. Moreover, rBCG::XB elicited stronger and more persistent Ag85B-specific CTL activity. When compared to the BCG group, rBCG::XB provided significant protection against MTB infection, markedly reducing pulmonary pathological changes [151]. These results suggest that rBCG::XB has the potential to provide better and more persistent protection than BCG, warranting further evaluation as a tuberculosis vaccine candidate.

BCG::ESX-1Mmar is a low-virulence recombinant BCG strain engineered to express the Mycobacterium marinum ESX-1 region [152]. Preclinical studies have demonstrated that BCG::ESX-1Mmar induces antigen-specific multifunctional CD4^+^ Th1 cells and stimulates type I IFN and inflammasome activation, modulating the differentiation and function of CD8^+^ T cells. Compared to BCG, BCG::ESX-1Mmar provided significantly enhanced protection against MTB infection, with markedly reduced MTB counts in the lungs and spleens of vaccinated mice [152].

BCGΔBCG1419c is a candidate strain with a deletion in the *BCG1419c* gene, which encodes a c-di-GMP phosphodiesterase [153]. In a guinea pig model infected with MTB, BCGΔBCG1419c reduced the secretion levels of pulmonary TNF-α and IL-17. When compared to BCG, BCGΔBCG1419c significantly alleviated pulmonary bacterial loads and pathological changes in the lungs, liver, and spleens of guinea pigs [154]. In a mouse model, BCGΔBCG1419c induced PPD-specific CD4^+^ and CD8^+^ T cell responses and central memory T cells. Moreover, BCGΔBCG1419c significantly reduced bacterial loads in the lungs of mice compared to BCG [153]. These studies suggest that BCGΔBCG1419c could be a promising vaccine candidate.

#### Subunit vaccines

Subunit vaccines, which comprise defined antigens from MTB, have shown promising results in preclinical models due to their potential to induce targeted immune responses. This section will detail the immunogenicity and protective efficacy of several subunit vaccine candidates, highlighting their unique compositions and mechanisms of action.

1. CysVac2/Advax: a fusion protein-based subunit vaccine

The subunit vaccine candidate, CysVac2/Advax, consists of the fusion protein CysVac2 containing Ag85B and CysD (Rv1285) antigens, combined with the polysaccharide adjuvant Advax [155]. In a mouse model, CysVac2/Advax effectively induced antigen-specific Th1-type responses and multifunctional CD4 + T cells, leading to the secretion of cytokines such as IFN-γ, TNF, and IL-17A. Notably, in MTB-infected mice, the CysVac2/Advax group demonstrated reduced pulmonary bacterial loads and alleviated pathological changes compared to the BCG group, showcasing significant protective effects [155]. These findings position CysVac2/Advax as a strong candidate for further preclinical evaluation and potential human trials.

(2) LT70: a peptide-based subunit vaccine

The LT70 subunit vaccine, composed of peptides from ESAT-6, Ag85B, MTB8.4, MPT64 190–198, and Rv2626c antigens, along with the adjuvant DDA/Poly(I:C), has exhibited promising outcomes in a mouse model [156]. Compared to the BCG group, the LT70 group significantly induced high levels of IFN-γ and IgG1 and IgG2c antibodies, effectively reducing bacterial loads in the lungs and spleens of MTB-infected mice. The BCG priming-LT70 boosting immunization strategy further enhanced the protective effects of BCG by alleviating pulmonary pathological changes in mice [156, 157].

(3) CFMO-DMT: a multistage fusion protein subunit vaccine

CFMO-DMT is a multistage fusion protein subunit vaccine composed of four MTB antigens (Rv2875, Rv3044, Rv2073c, and Rv0577) and the strong inducer of Th1-type immunity DMT adjuvant. It is widely recognized by T cells from patients with LTBI and ATB [158, 159]. Preclinical studies have shown that CFMO-DMT induced the production of IFN-γ, IL-2, and TNF-α cytokines and IgG, IgG1, and IgG2a specific antibodies in mouse splenocytes. CFMO-DMT provided the same level of protection as BCG against MTB-infected mice in the lungs and spleens. The BCG priming-LT70 boosting group showed the best protective effects by eliminating MTB from the lungs and spleens of LTBI mice, preventing MTB reactivation [158].

(4) H64/H74/H107: subunit vaccines with multiple antigens

The H64/H74/H107 subunit vaccines, composed of multiple MTB antigens, have demonstrated varying degrees of protective efficacy. H64 (EsxA, EspC, EspD, EspF, EspR, and PE35), H74 (EsxA, EspA, EspB, EspC, EspD, and EspR), and H107 (EsxA, EspA, EspC, EspI, MPT64, MPT70, MPT83, and PPE68) have been evaluated in preclinical models [160, 161]. H64 has been shown to provide good protection against MTB infection in mice, reducing pulmonary bacterial loads and extending survival times. H74 induces low-differentiation CD4^+^ T cells (secreting TNF-α alone or in combination with IL-2), which are protective against pulmonary MTB infection. Compared to the BCG group, BCG priming-H74 boosting significantly reduced pulmonary bacterial loads in mice [160]. In a mouse model, co-administration of BCG and H107 enhanced the immunogenicity of both vaccines and the protective effects of BCG. H107 also significantly increased the clonal diversity of the CD4 + T cell repertoire induced by BCG, inducing low-differentiation CD4 Th1 cells and Th17-type immune responses [161].

(5) Multi-epitope vaccine (MEV): MP3RT

MEVs represent a cutting-edge approach in vaccine development, attracting considerable attention due to their capacity to target multiple antigenic determinants simultaneously. This strategy is designed to broaden the immune response and augment protection against pathogens, such as MTB. In our recent research, we have made significant progress by developing the first tuberculosis MEV, designated MP3RT, specifically tailored for the Chinese population. This vaccine was engineered using reverse vaccinology techniques, enabling the precise identification and selection of the most promising antigenic epitopes [22]. Our studies have elucidated the immunoprotective mechanisms of MP3RT, revealing a predominantly Th1-type T lymphocyte response, accompanied by a significant reduction in regulatory T cells (Treg) and Th2-type T lymphocytes [162]. This Th1-skewed response is pivotal for effective cell-mediated immunity against intracellular pathogens like MTB.

#### DNA vaccines

The DNA vaccine, *DNA-hsp65*, which encodes the heat shock protein 65 (hsp65) from *Mycobacterium leprae*, has demonstrated notable immunogenicity in preclinical models [163]. In murine studies, this vaccine candidate has been shown to elicit antigen-specific CD4 + and CD8 + T cell responses along with the production of IFN-γ. Notably, the CD8 + CD4 − /CD44hi T cell subset was identified as the most effective in providing protection, with the potential to maintain this efficacy for up to 15 months [164]. Comparative analyses have revealed that *DNA-hsp65* vaccination significantly decreased the bacterial burden in the lungs of mice, which is attributed to the induction of a Th1-skewed immune response, an increase in CD8 + T cells and γδ T cells, and a reduction in the Th17 cell subset [165]. Furthermore, Eduardo D C Gonçalves et al. reported that the sequential administration of BCG followed by *DNA-hsp65* as a boosting strategy synergistically enhanced the immune response and protection compared to BCG alone, as evidenced by increased secretion of IFN-γ, IL-12, IL-10, and IgG2a antibodies, as well as reduced bacterial loads and lung pathology in vaccinated mice [166]. These findings position *DNA-hsp65* as a robust candidate for further development in tuberculosis vaccine research.

#### mRNA vaccines

The advent of mRNA vaccine technology has opened new avenues in the field of vaccinology. One such candidate, *repRNA-ID91*, is based on the alphavirus replicon RNA backbone and encodes a fusion protein composed of four MTB antigens: esxV, RpfD, PPE60, and Ag85B [167]. Studies in murine models have indicated that a heterologous priming-boosting strategy using *repRNA-ID91* followed by *ID91* combined with GLA-SE adjuvant elicits superior immunogenicity and protective efficacy compared to homologous boosting regimens [167, 168]. Both *ID91* + GLA-SE and *repRNA-ID91* + NLC (nanoparticle lipid carrier) were found to induce IgG antibodies and CD4^+^ CD44^+^ Th1 T cells producing IFN-γ, IL-2, and TNF. However, the *ID91* + GLA-SE regimen induced a more robust CD4^+^ T cell response, while *repRNA-ID91* + NLC additionally triggered CD8^+^ CD44^+^ Th1 T cells expressing the same cytokines [167]. The *repRNA-ID91* priming-*ID91* + GLA-SE boosting strategy was also found to induce antigen-specific IgG, IgG1, and IgG2c antibodies along with CD4^+^ and CD8^+^ T cells in mice, and significantly reduced bacterial loads in both the lungs and spleens [168]. These results highlight the potential of *repRNA-ID91* as an effective tuberculosis vaccine candidate.

### Vaccines in clinical trials

**Fig. 5:**
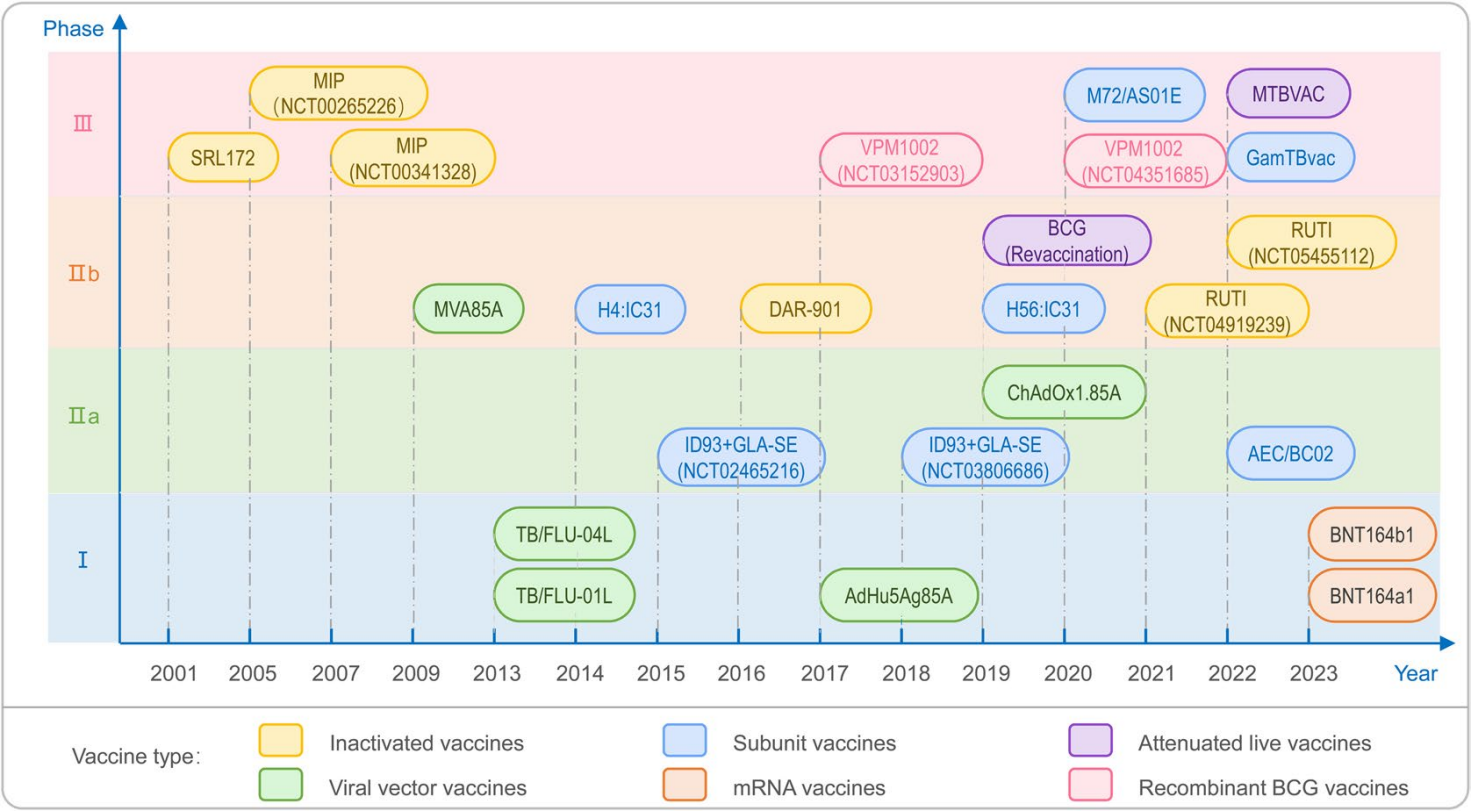
Timeline of TB vaccine candidates in clinical phases I-III. Vaccines are color-coded by type: Inactivated (yellow), subunit (blue), viral vector (green), mRNA (orange), and attenuated live (purple). Notable candidates include MIP, SRL172, MVA85A, and ChAdOx1.85A. Phases indicated on the y-axis, with years on the x-axis

With the deepening understanding of MTB genetic systems, proteomics, and immune mechanisms, we are facing new opportunities to develop safer and more effective TB vaccines [169]. A comprehensive TB vaccine strategy should cover three key objectives: preventing primary infection and subsequent disease, preventing reactivation of latent infection, and serving as an immunological adjuvant to standard TB treatment, thereby accelerating patient recovery [4, 18, 22, 170]. This study reviews novel TB vaccines currently in clinical trials, which can be categorized into six major types based on their technical characteristics: inactivated vaccines, attenuated live vaccines, recombinant BCG, subunit vaccines, viral vector vaccines, and mRNA vaccines (Table 2) (Fig. 5) [171–178]. These candidate vaccines each have their unique features, ranging from traditional whole-cell vaccines to precisely designed vaccines based on molecular biology, reflecting the extensive exploration and innovative progress in the field of TB vaccine research.

**Table 2:** Current landscape of tuberculosis vaccine candidates in clinical development

Vaccine	Vaccine Type	Vaccine Components and Characteristics	Manufacturer/Research Institution	Phase	NCT Number	Results	Refs
MIP	Inactivated	Made from inactivated *Mycobacterium indicus pranii*, unique immunogenicity	ICMR, CADILA	Phase III	NCT00341328 NCT00265226	NA At week 4, MIP group showed a significantly higher sputum culture conversion rate of 67.1% compared to the placebo group at 57% (p = 0.0002)	[171]
SRL172	Inactivated	Derived from non-tuberculous mycobacteria, safe and immunogenic in HIV-infected and uninfected individuals	DHMC, NIAID	Phase III	NCT00052195	SRL172 is safe and significantly protective against tuberculosis, with a confirmed TB risk ratio of 0.61 (*P* = 0.03), disseminated TB risk ratio of 0.52 (*P* = 0.16), and suspected TB risk ratio of 1.17 (*P* = 0.46)	[172]
DAR-901	Inactivated	Utilizes broth culture technique, improved scalability for commercial production	Dartmouth	Phase IIb	NCT02712424	The 1 mg DAR-901 three-dose series is safe but does not prevent tuberculosis infection (no IGRA conversion)	[173]
RUTI	Therapeutic	Composed of detoxified, fragmented MTB, delivered via liposomal system	Archivel Farma	Phase IIb	NCT04919239 NCT05455112	NA NA	
MTBVAC	Live Attenuated	Precise deletion of phoP and fadD26 genes, enhanced protection against MTB	Biofabri, S.L	Phase III	NCT04975178	NA	
BCG (Revaccination)	Live Attenuated	Long-term application and optimized distribution within health systems, for adolescent booster immunization	Gates MRI, Aeras, Sanofi Pasteur	Phase IIb	NCT04152161	NA	
VPM1002	Recombinant BCG	Replacement of urease C gene in BCG, enhanced activation of CD4 + and CD8 + T cells	SIIPL, VPM	Phase III	NCT03152903 NCT04351685	NA NA	
M72/AS01E	Subunit	Fusion protein of MTB39a and MTB32a with AS01E adjuvant, induces durable T cell responses	Gates MRI, GSK	Phase III	NCT04556981	NA	
GamTBvac	Subunit	Contains Ag85A and ESAT6-CFP10 fusion proteins, combined with DEAE-dextran nanoparticle adjuvant	Gamaleya Res. Centre, MoH Russia	Phase III	NCT04975737	NA	
H56:IC31 (AERAS-456)	Subunit	Ag85B-ESAT6-Rv2660c fusion protein with IC31 adjuvant, enhances immune response	SSI, Valneva, IAVI	Phase IIb	NCT03512249	NA	
H4:IC31 (AERAS-404)	Subunit	Ag85B-TB10.4 fusion protein with IC31 adjuvant, as BCG booster	SSI, Sanofi Pasteur, Valneva, Aeras	Phase IIb	NCT02075203	H4:IC31 shows significant immunogenicity, with a QFT conversion rate of 14.3%, a sustained conversion rate of 8.1%, and a vaccine efficacy of 30.5% (*P* = 0.16)	[174]
ID93 + GLA-SE (QTP101)	Subunit	Rv2608-Rv3619-Rv3620-Rv1813 fusion protein with GLA-SE adjuvant, enhances immune response	NIAID, NIH	Phase IIa	NCT03806686 NCT02465216	NA Compared to the placebo group, the two-dose series of 2 μg ID93 + 5 μg GLA-SE induced significant antigen-specific IgG and CD4 T-cell responses sustained for 6 months	[175]
AEC/BC02	Subunit	Ag85B and ESAT-6/CFP-10 fusion proteins with BC02 adjuvant, synergistic immune enhancement	Anhui Zhifei Longcom	Phase IIa	NCT05284812	NA	
MVA85A (AERAS-485)	Viral Vector	Modified Vaccinia Ankara virus expressing MTB Ag85A antigen	Aeras, University of Oxford	Phase IIb	NCT00953927	In the MVA85A group, 2% of infants developed tuberculosis with an incidence rate of 1.15/100 person-years; 13% of infants were infected with MTB	[176]
ChAdOx1.85A	Viral Vector	Replication-deficient chimpanzee adenovirus vector expressing MTB Ag85A antigen	University of Oxford	Phase IIa	NCT03681860	The ChAdOx1 85A prime-MVA85A boost vaccination regimen is safe and induces stronger Ag85A-specific IgG and IFN-γ responses than BCG revaccination	[177]
AdHu5Ag85A	Viral Vector	Based on replication-deficient human adenovirus type 5 vector expressing Ag85A antigen	McMaster University, CanSino	Phase I	NCT02337270	Both high and low doses of AdHu5Ag85A are safe and well-tolerated, inducing Ag85A-specific T-cell responses via aerosol and intramuscular administration; the low dose via aerosol notably induces multifunctional memory CD4 + and CD8 + T-cells in airway tissues	[178]
TB/FLU-01L	Viral Vector	Replication-deficient recombinant influenza virus expressing Ag85A	RIBSP Kazakhstan, SRII	Phase I	NCT03017378	NA	
TB/FLU-04L	Viral Vector	Recombinant attenuated influenza virus strain expressing ESAT-6 and Ag85A	RIBSP Kazakhstan, SRII	Phase I	NCT02501421	NA	
BNT164a1	mRNA	Multi-antigen vaccine based on mRNA technology, demonstrating potential of mRNA vaccines in TB prevention	BioNTech SE	Phase I	NCT05547464	NA	
BNT164b1	mRNA	Another multi-antigen vaccine based on mRNA technology, advancing mRNA vaccine research alongside BNT164a1	BioNTech SE	Phase I	NCT05547464	NA	

*Abbreviations: ICMR* Indian Council of Medical Research, *CADILA* Cadila Pharmaceuticals Ltd., *DHMC* Dartmouth-Hitchcock Medical Center, *NIAID* National Institute of Allergy and Infectious Diseases, *Gates MRI* Bill & Melinda Gates Medical Research Institute, *SIIPL* Serum Institute of India Private Limited, *VPM* Vakzine Projekt Management GmbH, *GSK* GlaxoSmithKline, Gamaleya Res. Centre, MoH Russia: Gamaleya Research Centre, Ministry of Health of Russia, *SSI* Statens Serum Institut, *IAVI* International AIDS Vaccine Initiative, *NIH* National Institutes of Health, *IDRI* Infectious Disease Research Institute, *RIBSP* Research Institute for Biological Safety Problems, *SRII* Smorodintsev Research Institute of Influenza

#### Inactivated TB vaccines

Inactivated TB vaccines represent a conventional approach in the prevention and treatment of TB. These vaccines are produced by inactivating MTB through physical or chemical means, effectively eliminating its pathogenicity while preserving crucial immunogenic properties [6]. Vaccines developed using this methodology utilize either whole-cell MTB or its lysed components to elicit immune responses against multiple MTB antigens [6]. Historically, inactivated vaccines have played a significant role in TB control efforts [6]. The primary mechanism of action for these vaccines involves the stimulation of Th1 cell-mediated immune responses and humoral immunity, which are effective in combating extracellular MTB infections and have demonstrated efficacy in TB control [17, 18]. Inactivated vaccines offer a notable advantage in their application due to their high safety profile. However, their immunogenicity may be comparatively lower than that of attenuated live vaccines, such as BCG. To enhance the immunological efficacy of inactivated vaccines, researchers are investigating various strategies, including refinement of vaccine preparation processes and the incorporation of immunological adjuvants. Several inactivated TB vaccine candidates have progressed to clinical trial stages, including MIP, SRL172, DAR-901, RUTI, and Vaccae. The ongoing research and development of these vaccines offer promising prospects for TB control and may play a crucial role in future immunization strategies against the disease.

1. MIP

*Mycobacterium indicus pranii* (MIP), a vaccine based on heat-killed *Mycobacterium W*, was initially developed for leprosy treatment but has shown potential in TB prevention. MIP contains unique immunogens and abundant members of the PE/PPE protein family found in MTB [179, 180], which activate both innate and adaptive immune responses. MIP promotes Th1 and Th17 immune responses while suppressing the Th2 pathway, leading to the activation of macrophages and dendritic cells [181]. Preclinical studies in murine TB models demonstrated that MIP, administered intranasally in both live and inactivated forms, provided significant protection [182–184]. Notably, intranasal administration not only reduced pulmonary MTB burden but also improved lung pathology. In the 1990s, a randomized, placebo-controlled clinical trial in India further confirmed MIP's efficacy in TB prevention [185]. A 10–13 year follow-up study of leprosy patients' household contacts vaccinated with MIP showed a significant reduction in TB incidence compared to the placebo group [185]. Based on these promising results, further research on MIP as a TB vaccine candidate progressed. A randomized, double-blind, placebo-controlled, multicenter phase III clinical trial (NCT00265226) conducted in India evaluated the efficacy and safety of MIP in category II TB patients [171]. Results demonstrated that MIP was not only well-tolerated with no adverse reactions but also significantly increased sputum culture conversion rates compared to placebo after four weeks of treatment, confirming MIP's ability to eliminate MTB in patients. These findings underscore MIP's potential as both a preventive and therapeutic vaccine for TB, warranting further investigation into its mechanisms of action and optimal administration protocols.

(2) SRL172

SRL172 is an innovative inactivated whole-cell vaccine derived from non-tuberculous mycobacteria (NTM), designed to enhance immune responses against TB. This vaccine has demonstrated favorable safety and immunogenicity profiles in both human immunodeficiency virus (HIV)-uninfected and HIV-infected populations [186]. Initial phase I and II clinical trials established SRL172's safety and ability to elicit immune responses through multiple injections. Building upon these promising results, a large-scale phase III clinical trial was conducted in Tanzania over a seven-year period, involving 2,013 participants. This randomized, double-blind, placebo-controlled study aimed to evaluate the safety, immunogenicity, and protective efficacy of SRL172 as a five-dose booster vaccine in HIV-infected patients previously immunized with BCG [172, 186–188]. The trial outcomes revealed that SRL172 was not only safe and well-tolerated in this population but also exhibited significant immunogenicity. Importantly, the vaccine provided additional protection against TB for both previously MTB-infected and uninfected patients.

These characteristics position SRL172 as a significant candidate in TB vaccine research, offering valuable scientific evidence for potential future TB prevention strategies. The vaccine's ability to boost immunity in HIV-infected individuals, a population particularly vulnerable to TB, underscores its potential importance in comprehensive TB control efforts. Further research may focus on optimizing dosing schedules, investigating long-term protection, and assessing efficacy in diverse populations to fully elucidate SRL172's potential role in global TB prevention strategies.

(3) DAR-901

DAR-901 is an inactivated whole-cell NTM vaccine, representing an innovative production method based on the SRL172 vaccine, utilizing the same master cell bank [173]. Unlike SRL172's agar cultivation, DAR-901 employs broth culture, an improvement that enhances scalability for commercial production [173]. Preclinical studies have demonstrated DAR-901’s potential as a BCG booster for TB prevention [189]. A phase I clinical trial conducted in the United States showed that DAR-901, administered as a three-dose series, was safe and immunogenic in BCG-immunized adults, regardless of prior MTB infection status, and was also safe in HIV-infected patients [190, 191]. A subsequent phase IIb trial in Tanzania involved BCG-vaccinated adolescents. Results indicated that a 1 mg dose of DAR-901 was safe and well-tolerated. Although it did not prevent initial or sustained IGRA (Interferon-γ Release Assay) conversion, DAR-901 vaccination enhanced immune responses to ESAT-6 [173]. The efficacy of DAR-901 as a TB prevention vaccine requires further validation through phase III clinical trials [173].

(4) RUTI

RUTI is a therapeutic vaccine composed of detoxified and fragmented MTB, delivered via a liposomal system [192]. Animal studies have shown that RUTI induces a mixed Th1/Th2/Th3 multi-antigenic immune response without observed local or systemic toxicity [193]. In MTB-infected mouse and guinea pig models, RUTI significantly reduced lung bacterial load and granulomatous infiltration compared to BCG [194]. A phase I clinical trial in 2007 evaluated RUTI's safety and immunogenicity in healthy volunteers at four different doses (5 μg, 25 μg, 100 μg, and 200 μg) [195]. Results showed some local indurations at injection sites, possibly related to inactive excipients. Nevertheless, RUTI successfully induced specific humoral and cellular immune responses [195]. A subsequent phase II trial in South Africa assessed RUTI's safety, tolerability, and immunogenicity in HIV-positive and negative LTBI patients [196]. RUTI demonstrated good safety and tolerability. HIV-negative patients showed good immune responses after 5 μg or 25 μg doses, with enhanced responses after the second dose. However, HIV-positive patients receiving 25 μg or 50 μg RUTI showed multi-antigenic immune responses after the first dose but no further enhancement after the second dose, possibly due to reduced CD4 + T lymphocyte counts in HIV-positive patients [196]. Currently, two ongoing phase IIb clinical trials (NCT04919239 and NCT05455112) aim to investigate RUTI as an adjunct to TB chemotherapy and compare RUTI immunotherapy with conventional treatment in terms of efficacy and safety in TB patients.

(5) *Mycobacterium vaccae*

*Mycobacterium vaccae* (*M. vaccae*) was initially isolated from the udder of cows by Boenickse R and Juhasz, and it is a rapidly growing non-pathogenic mycobacterium [197]. Studies have shown that *M. vaccae* can function by enhancing Th1-type immune responses and suppressing Th2-type immune responses. In mouse models, *M. vaccae* induces the production of CD8^+^ T cells and IFN-γ, and stimulates the secretion of cytokines such as interleukin-12 (IL-12) by CD8^+^ T cells and macrophages [198]. Phase I and II clinical trials conducted in Zambia and Finland demonstrated that a five-dose regimen of *M. vaccae* has good safety and immunogenicity, and is considered a candidate vaccine for preventing tuberculosis in HIV-infected individuals [186, 188]. A randomized, placebo-controlled, double-blind Phase III clinical trial conducted in Tanzania showed that the five-dose *M. vaccae* regimen is safe and provides a 39% protective effect against tuberculosis in HIV patients with a CD4 cell count of at least 200 cells/μl after the initial BCG vaccination [172].

In 1999, the Chinese Institute for the Control of Pharmaceutical and Biological Products collaborated with the Eighth Medical Center of PLA General Hospital to improve *M. vaccae* and named it Vaccae™. This product is manufactured by AnHui Zhifei Longcom Bio-pharmaceutical Co., Ltd. (now AnHui Zhifei Biological Products Co., Ltd.), and has the advantages of enhancing immunity, bidirectional regulation of immune responses, stimulating phagocytosis, and reducing pathological damage. It has been approved for the clinical adjuvant treatment of TB. Vaccae™ is the only TB immunotherapy drug recommended by the WHO [199]. Furthermore, a randomized, double-blind, placebo-controlled Phase III clinical trial (NCT01979900) was conducted in Guangxi, China, aimed at evaluating the preventive effect and safety of Vaccae™ in high-risk populations for TB infection. The trial has been completed, but its results have not yet been published.

#### Attenuated live vaccines

Attenuated live vaccines are developed using pathogens that have undergone multiple passages in laboratory conditions to reduce virulence, or live strains that closely resemble the original pathogen but exhibit enhanced safety profiles [4, 18, 200]. These vaccines contain active microorganisms that simulate the activation of the immune system during natural infection, eliciting robust and long-lasting immune responses. Most attenuated live vaccines require only one or two doses to confer lifelong immunity. Compared to inactivated vaccines, attenuated live vaccines demonstrate significant advantages, including enhanced immunogenicity and more durable protective effects. They persist in the human body for extended periods, continuously stimulating the immune system to produce antibodies and providing long-term protection [7]. Furthermore, attenuated live vaccines induce a broader immune response than subunit vaccines and typically do not require additional adjuvants to enhance immunogenicity [201]. Currently, MTBVAC and BCG (revaccination) are two attenuated TB vaccines undergoing clinical trials. However, it is important to note that while attenuated live vaccines are generally safe for most populations, they may pose risks for immunocompromised individuals, such as those with chronic illnesses, organ transplant recipients, and pregnant women, due to the presence of small amounts of weakened live pathogens.

1. MTBVAC

MTBVAC, a prominent candidate for TB vaccination, is currently in pivotal Phase III clinical trials [202]. Its attenuation strategy involves precise deletions of the phoP and fadD26 genes in MTB, conferring PhoP/PDIM deficiency while retaining MTB-specific antigens absent in BCG vaccines [203]. MTBVAC vaccination activates human monocytes through epigenetic reprogramming, inducing innate immune responses and demonstrating effective protection against lethal pneumonia in mouse models [204]. Animal studies have further confirmed MTBVAC's significant advantage over BCG in protecting rhesus macaques against MTB challenge [205]. MTBVAC is primarily targeted for neonatal vaccination but is also considered as a booster for adolescents and adults [202]. Phase Ia clinical trials in adults have established MTBVAC's safety profile, with no serious adverse events reported [206]. Phase Ib trials in neonates from high TB-burden countries have shown MTBVAC to be as safe as BCG with enhanced immunogenicity [207]. A Phase Ia/IIb trial (NCT02933281) initiated in South Africa in 2018 aims to determine MTBVAC dosage and assess its safety and immunogenicity in BCG-vaccinated adults. Although results are pending, the study's progression indicates recognition of MTBVAC's potential. In 2019, another Phase IIa trial (NCT03536117) commenced in South Africa to determine appropriate neonatal dosages and evaluate MTBVAC's safety and immunogenicity. Currently, a large-scale randomized, double-blind, controlled Phase III clinical trial (NCT04975178) is underway in sub-Saharan African TB-endemic regions to assess MTBVAC's efficacy, safety, and immunogenicity in neonates with and without HIV exposure. The outcomes of these trials will provide definitive evidence for MTBVAC's potential as a novel TB vaccine.

(2) BCG (Revaccination)

BCG vaccination, widely used for early childhood TB prevention, has been effective in preventing severe TB and reducing mortality in infants and young children [208]. While BCG efficacy varies in adults, its impact on children is significant. TB infection risk fluctuates with age, with school-age children having lower risk compared to infants, but risk increases again during adolescence [209]. This phenomenon suggests adolescence as a critical period for supplementing and optimizing vaccination strategies [210]. BCG revaccination leverages its long-term application and optimized distribution within health systems [211]. Studies indicate that BCG revaccination elicits positive immune responses with moderate safety profiles [211]. A Phase II clinical study in South Africa demonstrated 45.4% efficacy of BCG revaccination in reducing sustained QuantiFERON-TB Gold (QFT) conversion rates among adolescents, suggesting potential in interrupting TB infection processes [212]. To further evaluate the efficacy, safety, and immunogenicity of BCG revaccination, a randomized, observer-blind, placebo-controlled Phase IIb trial (NCT04152161) was initiated in South Africa in 2019. This study aims to assess the potential benefits of BCG revaccination in healthy children and adolescents, providing scientific evidence for future vaccination strategies.

#### Recombinant BCG

Recombinant BCG (rBCG) vaccines have been developed to enhance the protective efficacy of the existing BCG vaccine in adults, addressing its inconsistent effectiveness across different populations [7]. Advancements in modern molecular biology techniques have enabled genetic modifications of BCG, allowing for the insertion of exogenous genes into bacterial or viral vectors to construct rBCG vaccines [213]. The development of improved rBCG strains primarily employs two strategies: gene knockout and gene knock-in [214].

VPM1002 represents a prominent example of rBCG vaccines, achieved through genetic modification by replacing the urease C gene in BCG with the Hly gene from Listeria monocytogenes [215–217]. This modification significantly enhances VPM1002's capacity to activate CD4 + T and CD8 + T cells, promoting autophagy, inflammasome activation, and apoptosis, while effectively inducing Th1 and Th17 type immune responses [218–220]. A Phase II clinical trial conducted in South Africa demonstrated VPM1002's favorable safety profile and immunogenicity in HIV-unexposed neonates, eliciting CD4 + and CD8 + T cell responses comparable to those induced by BCG [221].

Currently, two Phase III clinical trials (NCT03152903 and NCT04351685) are underway to evaluate VPM1002's efficacy and safety in preventing TB recurrence and neonatal TB infection. The outcomes of these studies will provide crucial evidence for the further development and application of rBCG vaccines.

#### Subunit vaccines

Subunit TB vaccines represent an advanced vaccine development strategy, comprising immunogenic components isolated and purified from MTB, including proteins, polysaccharides, or peptides [7]. This purification approach confers higher purity to subunit vaccines compared to traditional vaccines while reducing components that may suppress immune responses [222]. Due to the absence of intact pathogens, subunit vaccines offer significant safety advantages and are considered safer than inactivated vaccines [222], making them one of the most attractive vaccine types in current research [223]. Additional benefits include cost-effectiveness, ease of production, and good stability [29]. However, subunit vaccines face limitations in eliciting broad immune responses due to their limited antigen content, potentially resulting in insufficient immunogenicity for long-term protection and relatively low memory immune capacity [7]. To overcome these limitations, subunit vaccines typically require adjuvants to enhance immunogenicity and promote immune protection or treatment [7]. Adjuvants, as multifunctional excipients, play a crucial role in improving immune response types, reducing required antigen quantities and vaccination frequencies, and enhancing vaccine stability and safety [224]. Currently, six protein subunit vaccines are undergoing clinical trials: M72/AS01E, GamTBvac, H56:IC31 (AERAS-456), H4:IC31 (AERAS-404), ID93 + GLA-SE(QTP101), and AEC/BC02. The results of these clinical trials will provide essential information for the application of subunit vaccines in TB prevention and treatment.

1. M72/AS01E 

The M72/AS01E candidate vaccine is an innovative TB subunit vaccine combining two immunogenic proteins from MTB, MTB39a and MTB32a, forming the M72 recombinant fusion protein, and combined with the AS01E adjuvant [225]. This vaccine has demonstrated the ability to induce specific lymphocyte proliferation and IFN-γ production in ATB patients and LTBI individuals [130, 134, 226]. M72/AS01E has shown good safety and reactogenicity in clinical trials, capable of eliciting persistent and effective CD4 and CD8 T cell responses, as well as CD4 + T cell-dependent IFN-γ recall responses [227]. A phase IIb randomized, double-blind, placebo-controlled clinical trial conducted in Kenya, South Africa, and Zambia evaluated M72/AS01E's protective efficacy against adult pulmonary tuberculosis (PTB). Results demonstrated good safety and a 54% protection rate against MTB infection in adults [225]. A three-year follow-up further confirmed M72/AS01E's protective efficacy at 49.7%, effectively preventing the progression from latent infection to active TB [228]. Currently, M72/AS01E is undergoing a phase II clinical trial (NCT04556981) in South Africa to assess its safety and immunogenicity in HIV patients receiving antiretroviral therapy.

(2) GamTBvac

The GamTBvac vaccine is a composite vaccine comprising MTB antigens Ag85A, ESAT6, and CFP10, non-covalently bound to dextran-binding domains (DBD) and combined with DEAE-dextran nanoparticle adjuvant containing CpG oligodeoxynucleotides (a TLR9 agonist) [229]. In mouse and guinea pig TB models, GamTBvac demonstrated strong immunogenicity and significant protective effects against the H37Rv strain [229]. A phase I clinical trial in Russia evaluated GamTBvac's safety and immunogenicity in BCG-vaccinated healthy adults. Results showed acceptable safety and good tolerability, with the ability to induce various cytokine secretions, with the half-dose vaccine group exhibiting the highest immunogenicity [90]. Subsequent phase II clinical trials further confirmed GamTBvac's safety and immunogenicity in BCG-vaccinated healthy volunteers, demonstrating induction of antigen-specific INF-γ release, expression of Th1 cytokines by CD4 + T cells, and IgG responses [230]. Based on these positive results, a phase III clinical trial (NCT04975737) has been initiated to assess GamTBvac's safety and efficacy in preventing PTB among HIV-uninfected individuals aged 18 to 45 years.

(3) H56:IC31 (AERAS-456)

H56:IC31 (AERAS-456) is a subunit vaccine combining the H56 protein with the IC31 adjuvant [192]. The H56 protein is a hybrid protein composed of latency-associated protein Rv2660c, Ag85B, and ESAT6, designed to significantly enhance protection against MTB infection in mice [231]. The IC31 adjuvant, a dual-component adjuvant containing antimicrobial peptide (KLK) and TLR9 agonist ODN1a [232], has been shown to enhance vaccine immunogenicity. In a Phase I randomized trial, H56:IC31 demonstrated acceptable safety in adults who recently completed treatment for drug-sensitive pulmonary TB and induced predominantly CD4 + T cell responses [233]. A Phase Ib randomized placebo-controlled trial in South Africa further confirmed the safety and immunogenicity of H56:IC31 in BCG-vaccinated, HIV-negative healthy adolescents, eliciting robust CD4 + T cell responses and serum IgG [212]. An ongoing Phase IIb double-blind, randomized, placebo-controlled trial (NCT03512249) aims to evaluate the safety and efficacy of H56:IC31 in reducing TB recurrence rates among HIV-negative adults successfully treated for drug-sensitive PTB.

(4) H4:IC31 (AERAS-404)

H4:IC31 (AERAS-404) is a subunit candidate vaccine composed of antigens Ag85B and TB10.4, combined with the IC31 adjuvant [234]. This vaccine is primarily studied as a booster for BCG. Preclinical studies have shown that H4:IC31 can induce antigen-specific CD4 T cells producing IFN-γ, IL-2, and TNF-α in humans [235, 236]. A Phase Ib randomized clinical trial in Cape Town, South Africa, evaluated the safety and immunogenicity of H4:IC31, H56:IC31, and BCG revaccination. Results indicated that all three vaccinations were safe, well-tolerated, and immunogenic. Both H4:IC31 and H56:IC31 vaccines induced Ag85B-specific CD4 + T cell responses and H4- and H56-specific IgG antibodies, but no antigen-specific CD8 + T cell responses were detected [212]. A Phase II clinical trial conducted in 2014 further assessed the safety, immunogenicity, and efficacy of H4:IC31 and BCG revaccination in preventing MTB infection in adolescents [174].

(5) ID93 + GLA-SE (QTP101)

ID93 + GLA-SE (QTP101) is a subunit vaccine composed of four MTB antigens (Rv1813c, Rv2608, Rv3619c, Rv3620c) combined with the TLR4 agonist GLA-SE as an adjuvant [234]. In mouse models, this vaccine demonstrated high levels of IFN-γ, IL-2, and TNF-α secretion, significantly reducing bacterial loads in the lungs and spleen [237]. A Phase I clinical trial in the United States evaluated the safety and immunogenicity of ID93 antigen alone or in combination with the GLA-SE adjuvant in healthy adults. Results showed that both vaccination methods were safe and well-tolerated, eliciting vaccine-specific humoral and cellular responses. Notably, ID93 + GLA-SE vaccination induced higher levels of ID93-specific antibodies IgG1 and IgG3 [238]. Another Phase I trial in South Africa further confirmed the safety and immunogenicity of ID93 + GLA-SE in HIV-negative, BCG-vaccinated healthy adults [239]. Additionally, a Phase IIa trial in the Cape Town area of South Africa assessed the safety and immunogenicity of ID93 + GLA-SE at different doses and injection regimens in patients previously treated for TB [175]. A Phase IIa trial in Korea (NCT03806686) aims to evaluate the safety and immunogenicity of the ID93 + GLA-SE vaccine in HIV-negative, QFT-negative, and previously BCG-vaccinated healthy adults [240].

(6) AEC/BC02

AEC/BC02 is a subunit candidate vaccine composed of MTB antigens Ag85B and ESAT6-CFP10 fusion protein, combined with the BC02 adjuvant (consisting of aluminum-containing CpG) [81]. Studies have shown that the combined use of CpG and aluminum produces a synergistic effect, enhancing both Th1 and Th2 immune responses [240, 241]. In preclinical studies, AEC/BC02 demonstrated the ability to induce high frequencies of antigen-specific IFN-γ secreting T cells in mice, exhibiting significant cellular immune responses [87]. Although AEC/BC02 did not prevent MTB infection in a guinea pig prevention model, it effectively controlled MTB reactivation and reduced bacterial loads in lungs and spleen in a latent infection model, indicating potential as a therapeutic vaccine [87]. In 2017, a Phase I clinical trial (NCT03026972) was initiated to primarily assess the safety of AEC/BC02. Subsequently, in 2020, a single-center, single-dose, placebo-controlled Phase Ib clinical trial (NCT04239313) was conducted to evaluate the safety and immunogenicity of AEC/BC02 in healthy adults. The results of these two clinical trials have not yet been published. Furthermore, a double-blind, randomized, controlled Phase II clinical study (NCT05284812) is currently underway. This study aims to assess the safety, tolerability, and immunogenicity of the AEC/BC02 vaccine in individuals aged 18 and above with LTBI. This research will provide further clinical evidence for the efficacy of AEC/BC02 as a potential TB vaccine.

#### Viral vector vaccines

Viral vector vaccines utilize viruses as carriers to deliver and express protective antigens of MTB. These vaccines effectively mimic the natural infection process of pathogens, activating the immune system and potentially forming long-term immune memory. Viral vector vaccines are valued for their high safety, technological maturity, and robust immunogenicity. However, they also present potential drawbacks, including the risk of virulence reversion and instability of exogenous gene expression [7]. Additionally, vector-specific immune responses may interfere with subsequent vaccinations [242]. Nevertheless, viral vector vaccines under development aim to serve as preventive or post-exposure vaccines to enhance immune responses induced by BCG or MTB infection [242]. Currently, viral vector vaccines in clinical trials include MVA85A (AERAS-485), ChAdOx1.85A, AdHu5Ag85A, TB/FLU-01L, and TB/FLU-04L.

1. MVA85A (AERAS-485)

MVA85A (AERAS-485) is a viral vector vaccine based on the Modified Vaccinia Ankara strain, expressing MTB's Ag85A antigen. As a heterologous BCG booster, MVA85A demonstrated enhanced protection against animal TB infection in clinical trials [243]. A Phase I clinical trial in Cape Town, South Africa, showed good tolerability and immunogenicity in healthy adults, inducing sustained antigen-specific CD4 + T cell responses [244]. Phase II clinical trials further evaluated MVA85A's safety and immunogenicity in BCG-vaccinated healthy children and infants, demonstrating safety and induction of stable, durable, multifunctional CD4 and CD8 T cell responses [245]. A Phase IIb clinical trial in 2009 showed good tolerability and immunogenicity in BCG-vaccinated, HIV-uninfected healthy neonates, although its anti-TB efficacy remains unclear [176]. A 2018 Phase II trial assessed MVA85A's safety and immunogenicity in HIV-exposed neonates, showing safety and induction of early moderate antigen-specific immune responses [246].

(2) ChAdOx1.85A

ChAdOx1.85A is a TB candidate vaccine using a replication-deficient chimpanzee adenovirus vector expressing MTB's Ag85A antigen. Preclinical studies demonstrated that a single intranasal immunization of ChAdOx1.85A induced strong immune responses in mouse models [247]. A BCG prime-ChAdOx1.85A-MVA85A boost regimen (B-C-M) showed better protection than BCG alone [248]. A Phase I clinical trial evaluated the safety and immunogenicity of ChAdOx1.85A prime-MVA85A boost vaccination in healthy UK adults, demonstrating good tolerability and immunogenicity [249]. A 2018 Phase IIa trial in Uganda further assessed the ChAdOx1.85A–MVA85A regimen in BCG-vaccinated adolescents, comparing it with BCG revaccination, showing stronger immune responses for the ChAdOx1.85A–MVA85A regimen [177].

(3) AdHu5Ag85A

AdHu5Ag85A (formerly Ad5Ag85A) is a candidate vaccine based on recombinant human adenovirus type 5 (AdHu5) expressing MTB's Ag85A antigen [250]. In mouse models, AdHu5Ag85A induced strong antigen-specific T cell responses via intramuscular injection or airway mucosal immunization [251]. A 2013 Phase I trial evaluated AdHu5Ag85A's safety and immunogenicity in BCG-naive and previously BCG-vaccinated healthy adults, showing safety, good tolerability, and immunogenicity [252]. A Phase Ib trial in Canada further assessed AdHu5Ag85A's safety and immunogenicity via different administration routes, demonstrating safety and good tolerability for both routes [178].

(4) TB/FLU-01L

TB/FLU-01L, developed by the Research Institute of Influenza (Saint Petersburg, Russia), is an attenuated influenza strain Flu NS106 vaccine expressing MTB antigen ESAT-6 [253].Previous studies have shown the vaccine to be safe and immunotherapeutic in mouse models [253]. The vaccine was also safe and immunogenic in BCG-vaccinated adults [253]. A clinical trial conducted in BCG-vaccinated healthy adults found that intranasal or sublingual administration of the TB/FLU-01L TB vaccine was safe and well-tolerated [138]. The TB/FLU-01L vaccine induced antigen-specific immune responses in 72.2% of subjects in the sublingual administration group and 77.8% of subjects in the intranasal immunization group (NCT03017378) [138]. In 2017, a Phase I clinical trial in Kazakhstan aimed to evaluate TB/FLU-01L's safety and immunogenicity in BCG-vaccinated healthy adults, but results have not yet been published [4].

(5) TB/FLU-04L

TB/FLU-04L is a replication-deficient intranasal influenza vector vaccine expressing MTB antigens ESAT-6 and Ag85A, designed as a preventive booster for infants, adolescents, and adults [254]. Previous preclinical studies have shown that intranasal immunization of mice, rats, ferrets, monkeys, and rabbits with the TB/FLU-04L vaccine is safe and induces a protective immune response against virulent MTB [254–256]. A well-designed single-center, Phase I, double-blind, randomized controlled trial evaluated the safety and immunogenicity of the TB/FLU-04L TB vaccine in healthy adult subjects aged 18 to 50 years who had previously received the BCG vaccine (NCT02501421). The trial employed a two-dose vaccination regimen, administered on Day 1 and Day 21 of the study, and compared the results with a matched placebo group. Although the results of this pivotal study have not yet been publicly disclosed, it provides a crucial foundation for assessing the potential clinical value of the TB/FLU-04L vaccine.

#### mRNA vaccines

Following the successful development and widespread application of COVID-19 vaccines, mRNA vaccine technology has emerged as a frontier in vaccine research, demonstrating significant potential in the development of TB vaccines [257]. The underlying mechanism of mRNA vaccines involves introducing mRNA encoding specific antigens into host cells, leveraging cellular machinery to synthesize antigenic proteins, thereby eliciting an immune response and conferring protection against TB [258, 259].

A critical challenge in mRNA vaccine design and development is the selection of appropriate antigens with sufficient immunogenicity. Ideal vaccine antigens should induce protective immune responses, ensuring effective stimulation of immune protection against the pathogen [29]. mRNA vaccines exhibit several advantageous characteristics, including high safety profiles, potent immunogenicity, rapid development cycles, ease of large-scale production, and broad application prospects, thereby demonstrating significant value and potential in vaccinology and public health practices.

Currently, two TB mRNA vaccine candidates, BNT164a1 and BNT164b1, are undergoing Phase I clinical trials [260]. In 2022, a randomized, placebo-controlled, observer-blind, dose-finding Phase Ib/IIa trial (NCT05547464) was initiated to evaluate the safety and immunogenicity of these vaccines in BCG-vaccinated HIV-negative subjects and HIV-infected individuals. This trial aims to explore four distinct dose levels to determine the optimal dose that is both safe and tolerable within a three-dose injection regimen. Although trial results are pending, this study holds significant implications for the application of mRNA vaccines in TB prevention.

## TB therapeutic drug development

**Fig. 6:**
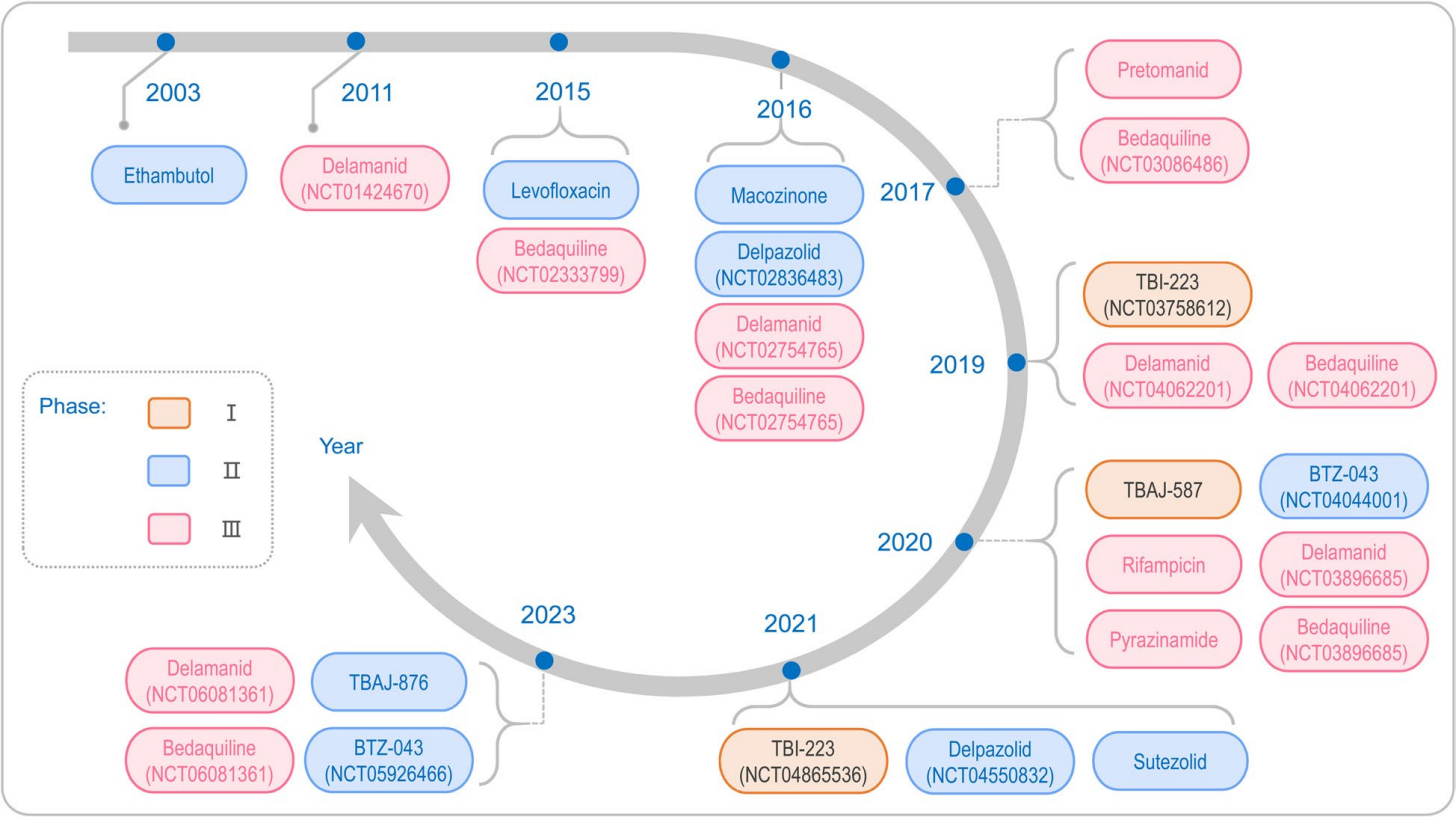
Timeline of anti-tuberculosis drug candidates in clinical development. Drugs are color-coded by phase: Phase I (orange), Phase II (blue), and Phase III (red). Years are marked on the timeline from 2003 to 2023, with drug names and NCT numbers annotated

MTB adopts alternate pathways in the host as they encounter a stressful environment that includes drugs and the host immune system to survive and become dormant [261]. Depending on the host environment, the MTB either causes active tuberculosis disease or becomes dormant, causing LTBI. The drug-susceptible (DR) TB can be treated using first-line drugs that include isoniazid (INH), rifampicin (RIF), pyrazinamide (PZA), and ethambutol** (**EMB). For treating LTBI, Disease Control and Prevention (CDC) recommends one course of rifapentine and RIF treatment for 3–4 months, and the second is INH monotherapy that lasts for 6–9 months in the US; the LTBI treatment may also contain PZA, EMB in many other countries [262]. The TB caused by MTB that is resistant to one or more of the first-line drugs [multidrug-resistant (MDR) and extensively drug-resistant (XDR)] requires treatment by second-line drugs, as shown in Table 3 [263–281]. Among them the important drugs include DLM, BDQ and pretomanid (PTM) [282]. Furthermore, we have identified the timeline for the progression of first-line and second-line anti-tuberculosis drugs into the latest clinical trial phases (Fig. 6).

**Table 3:** Anti-TB drugs with details of clinical trials of new drugs

Anti-TB drugs	Mode of action	Clinical trials	NCT Number	Safety of the drugs	References
**First-line drug for DS-TB**					
Isoniazid	Inhibits of MTB cell wall	Randomized clinical trial for LTBI			[263]
Rifampicin	Inhibits MTB DNA-dependent RNA synthesis	-		Well tolerated fever, gastrointestinal disturbances, and rashes	-
Pyrazinamide	Inhibition of protein synthesis	Phase 3, Randomized, Controlled Clinical Trial	NCT04856644	Variable tolerance	[264]
Ethambutol	Inhibition of cell wall synthesis	**phase 2 trial** for TB	NCT00140309	Well tolerated	[265]
**Second-line drug for MDR-TB**					
Levofloxacin	DNA gyrase inhibitor	Phase 2	NCT01918397		[266, 267]
Rifampicin (high dose)	RpoB inhibitor	Phase 3	NCT04485156	Well tolerated	[268, 269]
Delpazolid	Inhibition of protein synthesis	Phase 2	NCT02836483 NCT04550832		[270]
Sutezolid (Oxazolidinone)	Inhibition of protein synthesis	Phase 2	NCT03959566		[271]
TBI-223 (Oxazolidinone)	Inhibits the binding of N-formylmethionyl tRNA to ribosome	Phase 1	NCT03758612 NCT04865536		[272]
Delamanid (Nitroimidazole)	Inhibits cell wall synthesis	Phase 3, approved	NCT01424670 NCT02754765 NCT04062201 NCT03896685 NCT06081361	Well tolerated in adults	[273]
Pretomanid (Nitroimidazole)	Inhibits cell wall synthesis	Phase 3, approved	NCT03086486	Well tolerated	[274]
Bedaquiline (Diarylquinoline)	Inhibits mycobacterial ATP synthase	Phase 3, approved	NCT02333799 NCT02754765 NCT03086486 NCT04062201 NCT03896685 NCT06081361	Well tolerated but causes Nausea, arthralgia, headaches QTc interval prolongation	[275]
TBAJ-587 M (Diarylquinoline)	Inhibits mycobacterial ATP synthase and hERG potassium channel	Phase 1	NCT04890535		[276]
TBAJ-876 (Diarylquinoline)	Inhibits mycobacterial ATP synthase	Phase 2	NCT06058299		[277, 278]
Macozinone (Benzothiazinone)	DprE1 inhibitor	Phase 2	NCT03334734		[279, 280]
BTZ-043 (Benzothiazinone)	DprE1 inhibitor	Phase 2	NCT04044001 NCT05926466		[281]

### Properties of the anti-TB drugs

INH, which is a pro-drug which is activated by catalase-peroxidase of MTB leading to generation of reactive radicals of isonicotinoyl that react with NAD^+^ and NADP^+^ to form INH-NAD/(P) complex [283]. The INH, after the activation inhibits protein synthesis in MTB [284]. A mutation in target gene Inhibin Subunit Alpha is responsible for resistance of MTB for isoniazid. Rifampicin, a popular drug acts by interfering with protein synthesis by binding to RNA polymerase which is encoded by *rpoB* gene and mutations in this gene makes bacteria resistant to rifampicin [285]. EMB acts by interfering with the synthesis of the cell wall of the MTB. During the cell wall synthesis, EMB prevents polymerization of arabinogalactan upon action on EmbA and EmbB, the two membrane-associated arabinosyl transferases [286]. PZA acts on the MTB particularly when they are in macrophages during first 2 months of treatment thereby reducing the treatment duration [287]. The PZA is an analog of nicotinamide it needs to be converted into pyrazinoic acid by an enzyme PZase in MTB to be active in vivo [288]. A mutation in a gene pncA that encodes PZase makes MTB resistant to PZA [289]. Mutations in respective genes makes these drugs ineffective against the MTB and treatment by the second line anti-TB drugs.

### Second line anti-TB drugs that show promise in treating MDR-TB

BDQ is a new class of drug approved by Food and Drug Administration for the treatment of pulmonary MDR and XDR-TB [290]. Mechanism of action including binding of BDQ to c-ring of the F type ATPase of MTB and prevents synthesis of ATP [291, 292]. The advantage of BDQ is that this drug inhibits the ATP synthesis both in the cells of MTB that are actively dividing and also in LTBI but not the host cells [293]. This drug is currently in controlled phase II clinical trials and the results show that the BDQ is bactericidal and reduces treatment time significantly not just in people with DS-TB but also in MDR-TB [294]. DLM and Pretomanid are prodrugs and belongs to nitroimidazole family are used for treating MDR-TB [295]. These drugs have broad spectrum targets in MTB and acts by interfering with mycolic acid synthesis and cellular respiration. In patients with MDR-TB, DLM alone showed drug activity with in 14 days [296]. Among these drugs, the DLM shows good efficacy, least toxicity, and does not react with antiretroviral drugs making it suitable for treating MDR-TB, XDR-TB, and TB in HIV-positive individuals.

### Host-directed therapy

The symptoms of repeated infections of TB show the complex interaction between MTB and the host immune response [297]. The MTB and humans have co-evolved for centuries, establishing great cooperation between the host and pathogen; therefore, 90% of people infected with MTB do not develop clinical symptoms of TB [298, 299]. Suggesting how an immune response by the host tailors the outcome of the infection by MTB, which either leads to LTBI or active TB [300]. Despite the development of several anti-TB drugs, no effective treatment methods are available against LTBI and DR-TB. In addition, there is an emergence of MDR and XDR-TB, posing a major challenge to eradicating the disease. In addition to novel anti-TB drugs, we need alternate approaches to treat LTBI and MDR XDR-TB. Host-directed therapy (HDT) is an emerging approach for the treatment of TB. HDT is known to interfere with the host cell factors that MTB requires for persistence in the host and pathogenesis of the disease, to enhance protective immune responses against the MTB to reduce inflammation, and to balance the immune reactivity. The HDT is a novel strategy to improve the efficacy of TB treatment by using anti-TB drugs to modulate the immune response of the host for the MTB [301].

Improved knowledge about the pathogenesis of MTB and immunological mediators enables new host-directed approaches to treat TB using antibiotics. The HDT provides antimicrobial advantages through a) interplay of host responses used by MTB to persist and establish the TB disease in the host, b) enhances immune defense mechanism of the host against MTB, c) targeting pathways that contribute to immunopathology, and d) modulation of host factors that are associated with pathogenic responses [302, 303]. The HDTs are used to target the specific pathway that plays a crucial role in the pathogenesis of TB by MTB and to reduce the symptoms of such inflammation [302]. Many HDTs target highly conserved pathways of the host, and Table 4 details the important adjuvants used in HDT to treat TB [304–319]. In HDT, the adjuvants acclimatize the host immune cells to the presence of TB, improve antimicrobial activity, minimize the time needed to treat the TB, and reduce the inflammation and tissue damage. The HDT also minimizes exposure to TB drugs and prevents the development and/or spread of drug-resistant MTB. Treatment of TB using anti-TB drugs in combination with HDTs uses multi-mechanism treatment, allowing synergism and may require minimal amounts of the drugs, thus reducing the toxicity without affecting the treatment efficacy.

**Table 4:** Details of the adjuvants for enhancing the activity of anti-TB compounds

Adjuvant	Objective	Trials	Host target	Trial No	Reference
Azithromycin	Study the effect on TB patients	Phase 2		NCT03160638	[305]
Everolimus Auranofin Vitamin D3 CC-11050	Study safety and efficacy of the 4 adjuvants in comparison to rifabutin-modified TB therapy	Phase 2		NCT02968927	[305–308]
Vitamin D3	Study properties of Vit D3 after TB. Study the ability of Vit D3 to reduce the risk of acquisition of LTBI Study the properties of Vit D	Phase 2 Phase 3	Authophagy Via athelicidin	NCT03011580 NCT02880982	[309]
Vitamin D				NCT01992263	
Ibuprofen	Study the properties, safety, and efficacy of for the Treatment of XDR-TB	Phase 2		NCT02781909	[305]
N-acetyl cysteine	Study the sputum culture conversion and its effects	Phase 2		NCT03702738	[305]
Pravastatin	Study the safety, tolerability, and efficacy	Phase 2		NCT03456102	[305]
Acetylsalicylic acid (ASA) and Ibuprofen (IBU)	Study the adjunctive agents with standard anti-TB treatment compared with standard anti-TB treatment in DS-TB MDR-TB patients	Phase 2b		NCT04575519	[305]
Etoricoxib and TB vaccine (H56:IC31)	Study the safety and immune effect of the use of etoricoxib alone, H56:IC31 alone, and the combinatory use of both in addition to standard anti-TB drugs	Phase 1		NCT02503839	[305]
Metformin	Study the acid-fast bacillus sputum conversion ratio from positive to negative in pulmonary TB patients	Phase 1	Autophagy	NCT05215990	[310]
Metformin	Study the safety and tolerability in people with HIV and TB co-infection	Phase 2	Autophagy	NCT04930744	[310]
Lithium, prochlorperazine, valproic acid	-	-	Autophagy		[311]
Rapamicin	-	-	Authophagy		[312]
Statin	-	Phase 2	Cholesterol	NCT03882177	[313–315]
Aspirin	-	-	Eicosanoids	NCT04575519	[316]
Diclofenac	-	-	Eicosanoids		[317]
Enbrel	-	-	Granuloma		[318]
Bevacizumab	-	-	Granuloma		[319]

## Challenges and future directions in TB vaccine and therapeutic development

The development of effective TB vaccines faces a multitude of challenges, yet it also presents promising prospects for the future. This section will delve into the complexities and potential pathways to overcome these obstacles, offering insights into the current state of TB vaccine research.

### Overcoming antigen diversity and immune evasion mechanisms

The development of effective TB vaccines faces significant challenges due to the complex nature of MTB and its interaction with the human immune system [320, 321]. One of the primary obstacles is the diversity of antigens expressed by MTB during different stages of infection and disease progression [2]. This antigenic variation makes it difficult to identify a single or even a combination of antigens that can provide comprehensive protection against all forms of TB.

Furthermore, MTB has evolved sophisticated immune evasion mechanisms that allow it to persist in the host, even in the presence of a robust immune response [322]. These mechanisms include the ability to modulate host immune responses, alter antigen presentation, and survive within host cells. As a result, developing vaccines that can overcome these evasion strategies and induce long-lasting protective immunity remains a significant challenge.

To address these issues, future vaccine development efforts should focus on: (1) Identifying and targeting conserved antigens that are essential for MTB survival and virulence across different strains and stages of infection; (2) Developing multi-antigen vaccines that can induce broad-spectrum immunity against various MTB life cycle stages; (3) Investigating novel adjuvants and delivery systems that can enhance the immunogenicity of selected antigens and promote long-lasting immune responses; (4) Exploring strategies to target and neutralize MTB's immune evasion mechanisms, potentially through the inclusion of antigens involved in these processes. By addressing these challenges, researchers may be able to develop more effective vaccines that can provide robust protection against diverse MTB strains and overcome the pathogen's immune evasion tactics.

### Improving vaccine efficacy in diverse populations

Another significant challenge in TB vaccine development is ensuring efficacy across diverse populations with varying genetic backgrounds, environmental factors, and pre-existing immune conditions. The current BCG vaccine, while widely used, shows variable efficacy in different geographic regions and populations, highlighting the need for improved vaccine strategies that can provide consistent protection globally [208].

Several factors contribute to this variability in vaccine efficacy: (1) Genetic diversity: Different populations may have varying immune responses to specific antigens due to genetic polymorphisms [323]; (2) Environmental factors: Exposure to environmental mycobacteria and other pathogens can influence vaccine-induced immunity [324]; (3) Co-infections: The presence of other infections, particularly HIV, can significantly impact vaccine efficacy and safety [325]; (4) MTB infection status: MTB infection progresses through different stages, including latency and active disease. Populations can be divided into three categories: individuals with LTBI, those with ATB, and uninfected individuals. For those with LTBI, vaccines should aim to prevent progression to active TB by enhancing immune surveillance mechanisms that maintain the bacillus in a dormant state. In the case of ATB, vaccines may need to trigger a rapid and robust immune response to effectively control and eliminate the infection, which might require specific antigens or adjuvants that stimulate a strong Th1 response. For subclinical TB, where symptoms are mild or absent, vaccines should strike a balance between controlling the infection and minimizing immune-mediated tissue damage. For uninfected individuals, the primary goal of vaccination is prevention, and these vaccines should provide long-lasting immunity without causing adverse reactions. Vaccine development should be tailored to address these distinct population groups [174, 326].

To address these challenges, future research should focus on: (1) Conducting large-scale clinical trials in diverse populations to assess vaccine efficacy across different genetic backgrounds and environmental conditions; (2) Developing personalized vaccination strategies that take into account individual genetic and environmental factors; (3) Investigating the impact of co-infections and nutritional status on vaccine efficacy and exploring ways to optimize vaccination in these contexts; (4) Implementing post-vaccination surveillance programs to monitor long-term efficacy and identify potential factors affecting vaccine performance in different populations; (5) Designing targeted vaccines for specific population groups based on their MTB infection status (LTBI, ATB, subclinical TB, or uninfected) [327].

### Advancing novel vaccine platforms and delivery systems

As highlighted in the clinical trials section, various vaccine platforms are being explored for TB prevention, including subunit vaccines, viral vector vaccines, and mRNA vaccines. Each of these platforms offers unique advantages and challenges in terms of efficacy, safety, and practical implementation [2, 12, 326].

To further advance TB vaccine development, researchers should focus on: (1) Optimizing mRNA vaccine technology for TB: Building on the success of mRNA vaccines for COVID-19, further research is needed to adapt this technology for TB prevention [328, 329]. This includes improving mRNA stability, optimizing delivery systems, and identifying the most effective antigen combinations. (2) Enhancing subunit vaccine formulations: Continued efforts should be made to improve the immunogenicity of subunit vaccines through the development of novel adjuvants and delivery systems [330]. This may include exploring nanoparticle-based formulations or combination approaches with other vaccine platforms. (3) Refining viral vector vaccines: Further research into the selection of appropriate viral vectors and optimization of antigen expression could improve the efficacy of these vaccines. Additionally, addressing potential pre-existing immunity to viral vectors is crucial for ensuring consistent vaccine performance [331]. (4) Investigating novel delivery routes: Exploring alternative routes of administration, such as mucosal or intradermal delivery, may enhance vaccine efficacy by inducing more robust local immune responses at the primary site of MTB infection [332]. (5) Developing thermostable formulations: Creating vaccines that do not require cold chain storage would significantly improve their accessibility and implementation in resource-limited settings where TB is often endemic [333]. By advancing these novel platforms and delivery systems, researchers can potentially overcome some of the limitations of current TB vaccines and develop more effective, safe, and practical vaccination strategies for global implementation.

### Addressing the cost and accessibility of TB vaccines and drugs

Ensuring the affordability and accessibility of TB vaccines and drugs is crucial for effectively controlling the global TB epidemic, particularly in high-burden and resource-limited regions. Here are several key strategies to address this issue: (1) Cost-effective production strategies: The key to reducing costs lies in optimizing the large-scale production processes for vaccines and drugs. This includes increasing vaccine yield and reducing resource waste, thereby lowering the cost per dose. Promoting the production of generic TB drugs and vaccines can significantly reduce costs. Moreover, establishing partnerships with pharmaceutical companies in developing countries can not only facilitate this process but also enhance local production capabilities. (2) Development of thermostable formulations: Developing vaccine formulations that do not require a cold chain is essential for improving accessibility, especially in remote and resource-limited areas. Thermostable formulations can significantly reduce logistical costs and allow for wide distribution without the need for complex infrastructure, thereby expanding vaccine coverage. (3) Innovative funding and incentive mechanisms: Implementing funding strategies, including public–private partnerships, international funding initiatives, and government subsidies, can effectively alleviate the economic burden of TB treatment. Additionally, these mechanisms can incentivize research and development in TB therapeutics and vaccines. Implementing differential pricing strategies, adjusting prices based on the purchasing power of different countries or regions, is also an essential means of increasing affordability. (4) Regulatory support and global collaboration: Streamlining regulatory processes can expedite the approval and distribution of new TB drugs and vaccines. Coordinating regulations across countries through global health organizations such as the WHO and the Global Fund can accelerate access to new drugs and vaccines. International collaboration is critical to addressing the accessibility of TB drugs and vaccines, as it can facilitate resource sharing and create a unified response strategy to the TB epidemic. (5) Local production and capacity building: Establishing local production capabilities in high-burden countries can reduce dependence on imported drugs and vaccines, ensuring supply chain sustainability. Training local personnel in vaccine production and distribution can strengthen these countries' production and distribution capabilities. Technology transfer from developed to developing countries can improve local manufacturers' efficiency and reduce production costs, making TB drugs and vaccines more affordable. In summary, by addressing the issues in these areas, we can significantly improve the accessibility and affordability of TB vaccines and drugs, thereby enhancing global TB control efforts.

### Addressing the drug-resistance mechanism of persister mycobacterial cells

The MTB adopts many survival mechanisms when exposed to anti-TB drugs that include both genotypic resistance and phenotypic drug resistance. Genotypic drug resistance is caused by mutations in the genes that are drug-specific, as discussed earlier. Studies have shown that the bacteria develop drug-specific genotypic resistance due to single nucleotide polymorphism (SNPs) when the concentration of the drug is below the minimum inhibitory concentration (MIC); hence, maintaining the MIC level and adherence to the treatment regime needs to be followed [334]. Therefore, it is important to study the concentration-dependent emergence of resistance mechanisms to improve the drug development process and design novel regimens.

Phenotypic drug resistance includes all other resistance mechanisms that are not associated with any changes in the genes and is referred to as antibiotic persistence and tolerance [335, 336]. The tolerance to the drugs is non-specific and unrelated to any antibiotic class. It includes mechanisms such as drug efflux, cell wall permeability and biofilms, reduced growth rate, and changes in the metabolic rate of MTB. Interestingly, there are about 0.01–1% of an inherently heterogeneous population of bacteria that survive extensive treatment with antibiotics and are called antibiotic persisters. These “antibiotic persisters” are genetically drug-susceptible quiescent mycobacteria that survive exposure to antibiotics and stressful conditions in the host [335–338]. These persisting MTB cells become active when the antibiotic treatment is stopped, which leads to the relapse of TB [338]. Therefore, it is important to understand the characteristics and the precise mechanisms of the formation of persister cells. In-depth research at the molecular level to find the key components that are responsible for the formation and survival of the persister cell is critical to designing more effective treatment methods for persistent TB to prevent the relapse of TB.

## Future directions for TB vaccines and therapeutic research

As we advance in the fight against TB, innovative strategies are critical to overcoming the challenges posed by this disease. Future research directions in TB vaccines and therapeutics focus on several key areas: leveraging combination strategies for enhanced efficacy, exploring novel drug targets to counteract resistance, and employing personalized medicine to tailor treatments to individual patient profiles. These approaches aim to not only improve current treatment outcomes but also to address challenges such as drug-resistant TB and latent infections. By integrating insights from cutting-edge science and technology, including bioinformatics and AI, researchers are paving the way for more effective and tailored TB interventions.

### Combination strategies

Combination strategies play an important role in TB vaccine and therapeutic drug research. This approach involves the use of different vaccine candidates, drugs, or therapeutic methods together to enhance efficacy and address resistance issues. (1) Vaccine Combinations: Combination strategies aim to enhance immune responses by using different vaccine candidates in conjunction. For example, BCG vaccination can be used as a primary immunization alongside a booster with a subunit vaccine, leveraging each method’s advantages to increase the breadth and depth of immune responses [4, 234]. (2) Drug Combinations: In therapeutic drug research, combining drugs is a common strategy to improve treatment outcomes and reduce the development of resistance. By using various anti-TB drugs that target different stages of the MTB lifecycle, the efficacy of treatment can be enhanced. Furthermore, drug combinations can reduce the dosage required for individual drugs, thereby minimizing the risk of side effects.

### Developing anti-TB compounds targeting novel pathways of MTB

Anti-TB drugs developed earlier are effective against actively growing MTB, often require a long duration of treatment, and are toxic to the host and, hence, difficult in achieving the goal set by the WHO for combating TB. In addition, the clinically used drugs act on well-known targets of the MTB for which the bacterium has developed resistance, requiring the use of multiple drugs without much success [299]. Therefore, there is an urgent need for drugs that act on novel cellular pathways of MTB that have not been targeted earlier [299]. Sequencing of the MTB genome showed that MTB codes for four carbonic anhydrases (three β and one γ) (CAs) that are essential for the survival and pathogenesis of MTB in the host [299, 339, 340]. Some of the authors of this review have focused research on the CAs present in MTB for developing novel anti-TB compounds targeting these proteins. Both in vitro and in vivo studies in zebrafish have shown that these proteins are druggable targets for developing anti-TB compounds [341–343]. In addition, the drugs developed could have minimal or no off-target effect as humans do not contain β and γ CAs, unlike MTB. There is a potential to combine these drugs with first-line anti-TB drugs to reduce the duration of the treatment with the potential to treat LTBI and MDR-TB.

### Improving the HDT adjuvant therapy

There are several anti-TB drugs and host-directed therapies are available, and new drugs and HDTs are under different stages of clinical trials. However, there are still several challenges in the treatment of DR-TB and LTBI that include failure in diagnosis, lengthy treatment regimes because of non-adherence to the treatments, and constantly evolving MTB and evading the treatment methods. Currently, HDT adjuvants are used to treat TB and to achieve the desired results. However, there is very little information on the effective use of HDT adjuvants to promote immunological responses of the host to inhibit the growth of the pathogen. Similarly, few preclinical studies are available on immunomodulators, and this information is not enough to meet the needs of anti-TB drug development. There is a need for multiple screening methods to discover more immune modulators [344]. Before using immunomodulators to treat TB, several concerns need to be considered, such as the availability of pharmacokinetic data on the compounds, which include absorption, distribution, metabolism, and excretion, to help assess the drug-drug interaction for the treatment regime. No studies are available on the interaction of immunomodulators and first-line anti-TB drugs, and drug interaction mechanisms are not clear. Therefore, promising immunomodulators should be assessed to get the pracademics and toxicological data using suitable biological models for subsequent clinical trials.

There are several HDT adjuvants in clinical trials, but no guidelines are available for selection, dosage, application time, or treatment courses. In addition, there are no strategies for selecting an appropriate adjuvant that is safe and effective in combination therapy regimens. There is no agreement or criteria for the administration of adjuvants along with anti-TB drugs as a standard treatment, or such therapy can be customized to suit the individual patient. Furthermore, stakeholders should take into consideration the long-term harmful effects of adjuvants and the emergence of resistance.

To combat TB, it is important to minimize the duration of treatment regimens. To achieve this, we need appropriate treatment methods, including immunomodulators and anti-TB drugs [345]. In addition, it is important to choose the appropriate adjuvant to stimulate the host immune response and produce protective effects. The future of TB treatment depends on the advancement of HDT and corresponding anti-TB drugs to achieve personalized and multifunctional immunotherapy. For this to happen, we need further studies on novel immune activation and suppression products using innovative approaches that can contribute to advancement in the field. This can be done in collaboration with fields such as molecular biology, pharmacology, immunology, and clinical medicine to speed up clinical studies to collect clinical data for immunomodulators and identify new targets for HDT.

### Personalized medicine for TB treatment and prevention

Personalized medicine holds great potential for TB treatment and prevention. This approach involves tailoring treatment plans according to the patient’s specific genetic background, immune status, and disease phenotype. (1) Genotype-Based Treatment: By analyzing a patient’s genotype, responses to specific drugs can be predicted, enabling the selection of the most appropriate drugs and doses. For instance, certain genetic variations may influence drug metabolism, resulting in reduced or enhanced drug efficacy. Identifying these variations allows for personalized treatment regimens. (2) Immune Status Monitoring: TB vaccine research and development can also benefit from personalized medicine. By monitoring a patient’s immune status, the optimal timing and dosage for vaccination can be determined. For example, deep learning technologies can analyze immune status to predict the timing and strength of immune responses, thus optimizing vaccination strategies. (3) Therapeutic Vaccines: Therapeutic vaccines are a promising strategy aimed at treating patients already infected with MTB. These vaccines typically target specific antigens of the bacterium to activate the patient's immune system for infection clearance. For example, the RUTI vaccine, a non-live multi-antigen vaccine based on fragmented bacterial cell walls, has shown potential in controlling LTBI after short-term chemotherapy.

## Conclusions

The scourge of TB persists as a pivotal health crisis, with the disease's complex interplay of pathogenesis, immune evasion, and drug resistance posing formidable challenges to effective control and eradication. This comprehensive review has underscored the imperative for innovative approaches in TB vaccine development and therapeutic interventions, reflecting the dynamic and evolving landscape of TB research and treatment.

In the domain of vaccine development, while the BCG vaccine has served as the cornerstone of TB prevention for decades, its variable efficacy and limitations in protecting against adult pulmonary TB have highlighted the urgent need for new vaccines. The exploration of novel antigens, multi-antigen strategies, and advanced platforms such as mRNA technology presents a promising frontier, with candidates like M72/AS01E and ID93/GLA-SE demonstrating encouraging results in clinical trials. However, the path to a universally effective TB vaccine remains obstructed by the disease's antigenic diversity and immune evasion mechanisms. Future research must focus on identifying conserved antigens, enhancing vaccine immunogenicity through innovative adjuvants and delivery systems, and conducting extensive clinical trials to ensure broad efficacy across diverse populations.

The therapeutic landscape of TB is equally challenging, with the emergence of drug-resistant strains complicating treatment strategies. This review also presents information related to ant-TB drug development, including new drugs approved by the FDA and WHO for treating LTBI, MDR, and XDR-TB. We also discuss HDT to enhance the immunological response of the host towards the MTB pathogen, along with the information on anti-TB drugs and HDT immunomodulators in clinical trials.

In conclusion, this review has highlighted the critical advancements and enduring challenges in the development of TB vaccines and therapeutic drugs. It has emphasized the need for a concerted global effort to accelerate research, foster innovation, and implement effective strategies to combat this ancient foe. The prospects for a more effective TB vaccine and improved therapeutics are promising but require sustained investment, interdisciplinary collaboration, and a relentless pursuit of scientific breakthroughs. Only through such concerted efforts can we hope to turn the tide against TB and move closer to the ambitious goal of global TB elimination set forth by the WHO.

## Supplementary Information

Supplementary Material 1: Figure S1. Structural Prediction, Interactome, and Immune Response Profiling of Ag85A and Ag85B proteins. (a). 3D structure of Ag85A predicted by AlphaFold; (b). Proteins interacting with Ag85A; (c). Predicted cytokine response induced by Ag85A; (d). Predicted levels of antigen-special antibodies induced by Ag85A; (e). 3D structure of Ag85B predicted by AlphaFold; (f). Proteins interacting with Ag85B; (g). Predicted levels of antigen-special antibodies induced by Ag85B; (h). Predicted cytokine response induced by Ag85B.

Supplementary Material 2: Figure S2. The computationally predicted three-dimensional (3D) configurations, associated protein interactions, and the ensuing immunological responses for the PPE18 and Rv1813c proteins. 3D structure of PPE18 (a) and Rv1813c (e) predicted by AlphaFold, enumerates the proteins that are known to engage in molecular dialogue with PPE18 (b) and Rv1813c (f), predicted levels of antigen-special antibodies induced by PPE18 (c) and Rv1813c (g), predicted cytokine response induced by PPE18 (d) and Rv1813c (h).

## Data Availability

All data generated or analysed during this study are included in this published article.

## Abbreviations

AdHu5: Human adenovirus type 5
AI: Artificial intelligence
ATB: Active tuberculosis
BCG: Bacille Calmette-Guérin
BDQ: Bedaquiline
DLM: Delamanid
DR: Drug susceptible
EMB: Ethambutol
ESAT-6: Early Secretory Antigenic Target 6-kDa
HDT: Host-directed therapy
HIV: Human immunodeficiency virus
HSPs: Heat shock proteins
HspX: Heat Shock Protein X
IFN-γ: Interferon-γ
IL-10: Interleukin-10
IL-12: Interleukin-12
INH: Isoniazid
LTBI: Latent tuberculosis infection
MDR: Multi-drug resistance
MEV: Multi-epitope vaccine
MIP: *Mycobacterium indicus pranii*
MTB: *Mycobacterium tuberculosis*
NO: Nitric oxide
PBMC: Peripheral blood mononuclear cell
PPE: Proline-proline-glutamic acid
PTB: Pulmonary tuberculosis
PZA: Pyrazinamide
rBCG: Recombinant BCG
RD-1: Region of Difference 1
TB: Tuberculosis
TGF-β: Transforming growth factor-β
TLR: Toll-like receptors
TNF-α: Tumor necrosis factor-α
XDR: Extremely drug-resistant
