# Supplementary figures and images for: Tuberculosis vaccines and therapeutic drug: challenges and future directions
Source: Mol Biomed. 2025 Jan 22;6:4. doi: 10.1186/s43556-024-00243-6 (PMC11754781; doi:10.1186/s43556-024-00243-6)

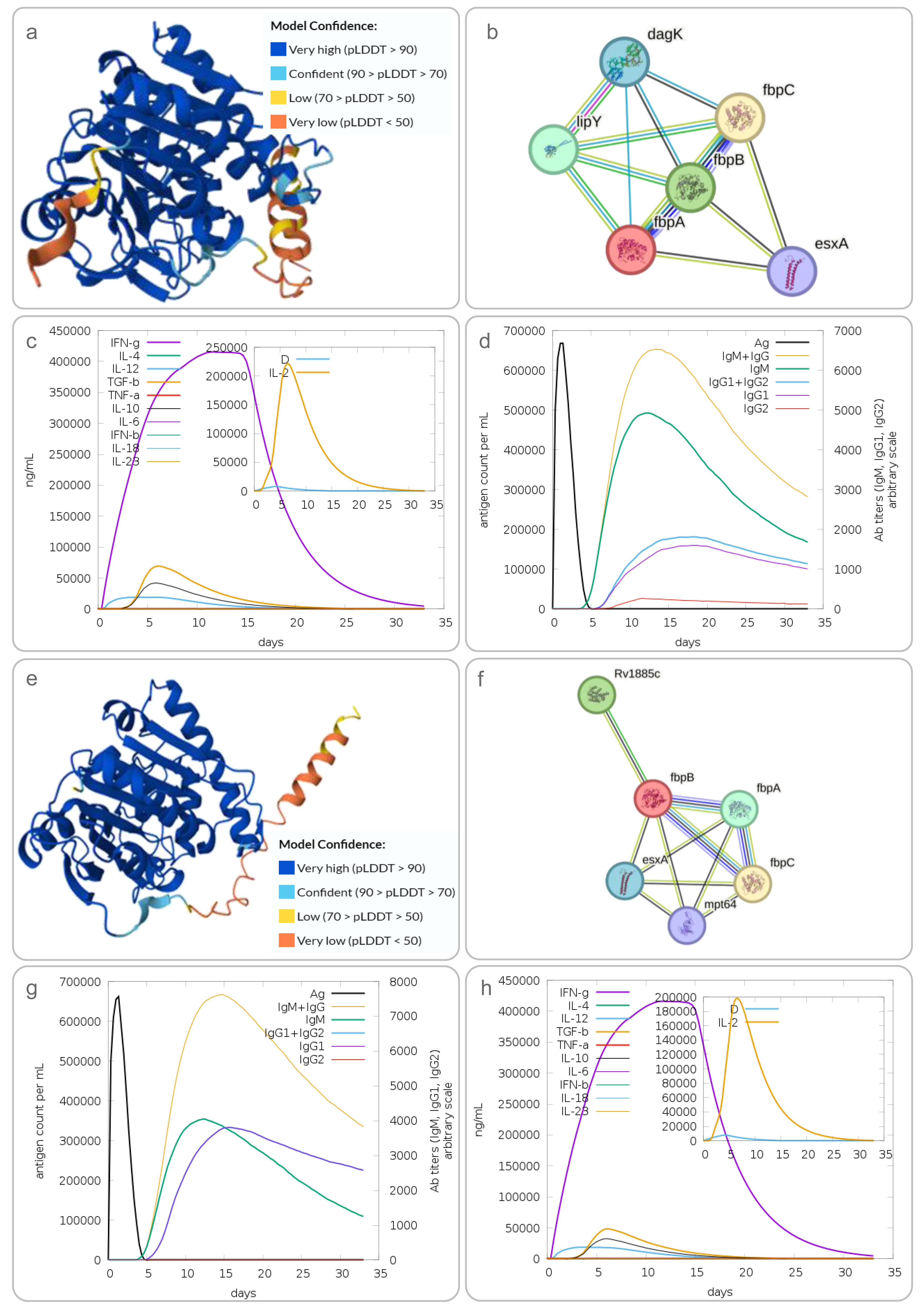

Supplement: Supplementary file 1 — Supplementary Material 1: Figure S1. Structural Prediction, Interactome, and Immune Response Profiling of Ag85A and Ag85B proteins. (a). 3D structure of Ag85A predicted by AlphaFold; (b). Proteins interacting with Ag85A; (c). Predicted cytokine response induced by Ag85A; (d). Predicted levels of antigen-special antibodies induced by Ag85A; (e). 3D structure of Ag85B predicted by AlphaFold; (f). Proteins interacting with Ag85B; (g). Predicted levels of antigen-special antibodies induced by Ag85B; (h). Predicted cytokine response induced by Ag85B. [file 43556_2024_243_MOESM1_ESM.jpg]

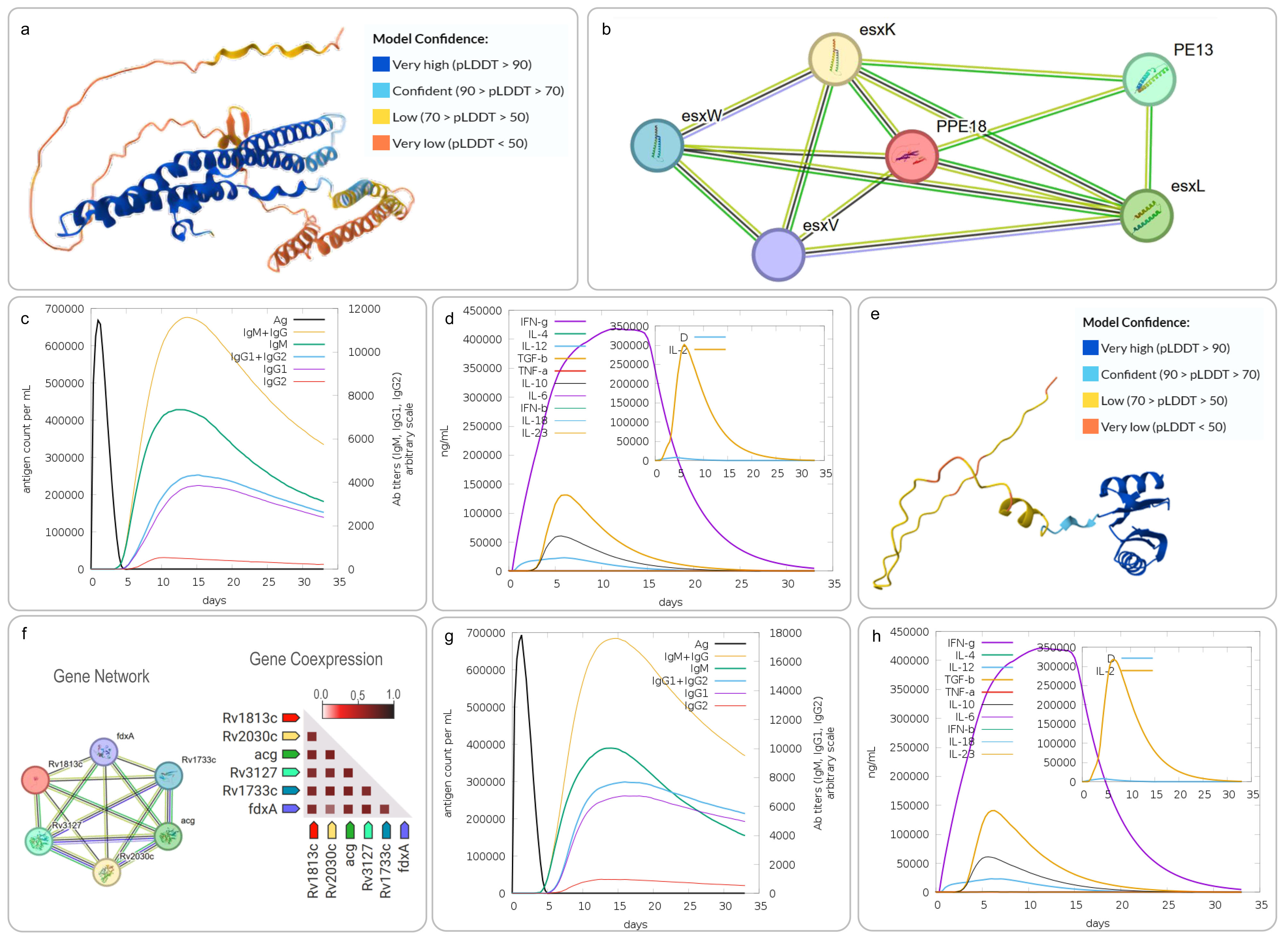

Supplement: Supplementary file 2 — Supplementary Material 2: Figure S2. The computationally predicted three-dimensional (3D) configurations, associated protein interactions, and the ensuing immunological responses for the PPE18 and Rv1813c proteins. 3D structure of PPE18 (a) and Rv1813c (e) predicted by AlphaFold, enumerates the proteins that are known to engage in molecular dialogue with PPE18 (b) and Rv1813c (f), predicted levels of antigen-special antibodies induced by PPE18 (c) and Rv1813c (g), predicted cytokine response induced by PPE18 (d) and Rv1813c (h). [file 43556_2024_243_MOESM2_ESM.jpg]
